# Astrocyte HIV-1 Tat Differentially Modulates Behavior and Brain MMP/TIMP Balance During Short and Prolonged Induction in Transgenic Mice

**DOI:** 10.3389/fneur.2020.593188

**Published:** 2020-12-15

**Authors:** Chaitanya R. Joshi, Satomi Stacy, Nathalie Sumien, Anuja Ghorpade, Kathleen Borgmann

**Affiliations:** ^1^Department of Microbiology, Immunology, and Genetics, University of North Texas Health Science Center, Fort Worth, TX, United States; ^2^Department of Pharmacology and Neuroscience, University of North Texas Health Science Center, Fort Worth, TX, United States

**Keywords:** HIV-associated neurocognitive disorders (HAND), neuroinflammation, TIMP1, iTat mice, anxiety, locomotor activity, tissue inhibitor of metalloproteinases 1

## Abstract

Despite effective antiretroviral therapy (ART), mild forms of HIV-associated neurocognitive disorders (HAND) continue to afflict approximately half of all people living with HIV (PLWH). As PLWH age, HIV-associated inflammation perturbs the balance between brain matrix metalloproteinases (MMPs) and their tissue inhibitors of metalloproteinases (TIMPs), likely contributing to neuropathogenesis. The MMP/TIMP balance is associated with cognition, learning, and memory, with TIMPs eliciting neuroprotective effects. Dysregulation of the MMP/TIMP balance was evident in the brains of PLWH where levels of TIMP-1, the inducible family member, were significantly lower than non-infected controls, and MMPs were elevated. Here, we evaluated the MMP/TIMP levels in the doxycycline (DOX)-induced glial fibrillary acidic protein promoter-driven HIV-1 transactivator of transcription (Tat) transgenic mouse model. The HIV-1 protein Tat is constitutively expressed by most infected cells, even during ART suppression of viral replication. Many studies have demonstrated indirect and direct mechanisms of short-term Tat-associated neurodegeneration, including gliosis, blood-brain barrier disruption, elevated inflammatory mediators and neurotoxicity. However, the effects of acute vs. prolonged exposure on Tat-induced dysregulation remain to be seen. This is especially relevant for TIMP-1 as expression was previously shown to be differentially regulated in human astrocytes during acute vs. chronic inflammation. In this context, acute Tat expression was induced with DOX intraperitoneal injections over 3 weeks, while DOX-containing diet was used to achieve long-term Tat expression over 6 months. First, a series of behavior tests evaluating arousal, ambulation, anxiety, and cognition was performed to examine impairments analogous to those observed in HAND. Next, gene expression of components of the MMP/TIMP axis and known HAND-relevant inflammatory mediators were assessed. Altered anxiety-like, motor and/or cognitive behaviors were observed in Tat-induced (iTat) mice. Gene expression of MMPs and TIMPs was altered depending on the duration of Tat expression, which was independent of the HIV-associated neuroinflammation typically implicated in MMP/TIMP regulation. Collectively, we infer that HIV-1 Tat-mediated dysregulation of MMP/TIMP axis and behavioral changes are dependent on duration of exposure. Further, prolonged Tat expression demonstrates a phenotype comparable to asymptomatic to mild HAND manifestation in patients.

## Introduction

During the antiretroviral therapy (ART) era, the brain remains a viral reservoir for HIV (1–3), and milder forms of HIV-associated neurocognitive disorders (HAND) affect nearly 18 million HIV-infected individuals lowering the quality of life (4–7). Patients suffering from these milder forms of HAND exhibit difficulty with working memory, executive functioning, and speed of information processing (6). Despite a mild or asymptomatic phenotype, complex underlying mechanisms are implicated in HAND pathogenesis. These mechanisms include secretion of proinflammatory mediators from infected and affected cells, blood-brain barrier (BBB) compromise, reactive astrogliosis, excitotoxicity, and imbalance of matrix metalloproteinases (MMPs) – tissue inhibitor of metalloproteinases (TIMPs) axis (8–10).

Imbalance of MMPs and TIMPs has been an important indicator of altered central nervous system (CNS) homeostasis. Increased MMPs disrupt the BBB *via* breakdown of tight junction proteins, recruit immune cells into the CNS, and cause direct neuronal damage potentially contributing to HAND pathology (11–13). On the other hand, MMP-independent neurotropic effects of TIMPs are well-documented (14–16), including our previous work on TIMP-1-mediated neuroprotection in primary human neurons in response to HAND-relevant stimuli (17). Four MMP and TIMP family proteins are the most investigated in the CNS due to their critical role(s) in modulating brain MMP/TIMP balance during multiple CNS diseases and disorders (18–20). These include both constitutively expressed (MMP-2 and TIMP-2) and inducible (MMP-9, TIMP-1) proteins following injury or inflammation.

In the context of HAND, levels of MMP-2 and/or MMP-9 were elevated in primary brain cell cultures treated with HIV or HIV-relevant stimuli (11, 21, 22) as well as in cerebrospinal fluid (CSF) specimens (23) and postmortem brain tissues of infected patients (24). Concurrently, reduced TIMP-1 levels were also observed in CSF and brain tissues of HIV-infected patients indicative of chronic inflammation (24). Our previous *in vitro* work in primary human astrocytes demonstrated that TIMP-1 increased or decreased with acute or prolonged HIV-relevant inflammatory stimuli, respectively (24, 25). It remains to be seen if such biphasic changes in TIMP-1 expression in response to acute vs. chronic inflammatory stimuli are observed *in vivo*, and it may provide insights on using TIMP-1 as a therapeutic option as its neurotropic effects are established.

To address this “knowledge gap,” we employed a doxycycline (DOX)-inducible, glial fibrillary acidic protein (GFAP) promoter-driven HIV-1 transactivator of transcription (Tat)-expressing transgenic (iTat) mouse model (26, 27). HIV-1 Tat is a key protein involved in neuronal dysfunction (28, 29), BBB disruption (22), oxidative stress (30, 31), elevating MMPs (32, 33), and possesses chemokine-like abilities that promote immune infiltration into the brain (26, 34). Several investigations using HIV-1 Tat based transgenic models elucidated effects of acute Tat expression, i.e., a few days after Tat induction (35–41), while the effects of prolonged Tat expression were seldom tested until recently (42, 43). It is imperative to test how duration of Tat expression alters its direct and indirect neurotoxic effects since Tat is known to be produced by infected cells during early as well as late stages, even in the presence of ART. More importantly, it would elucidate if TIMP-1 and subsequently MMP/TIMP axis could be modeled during acute versus prolonged HAND-relevant stimuli *in vivo*.

**Figure 1:**
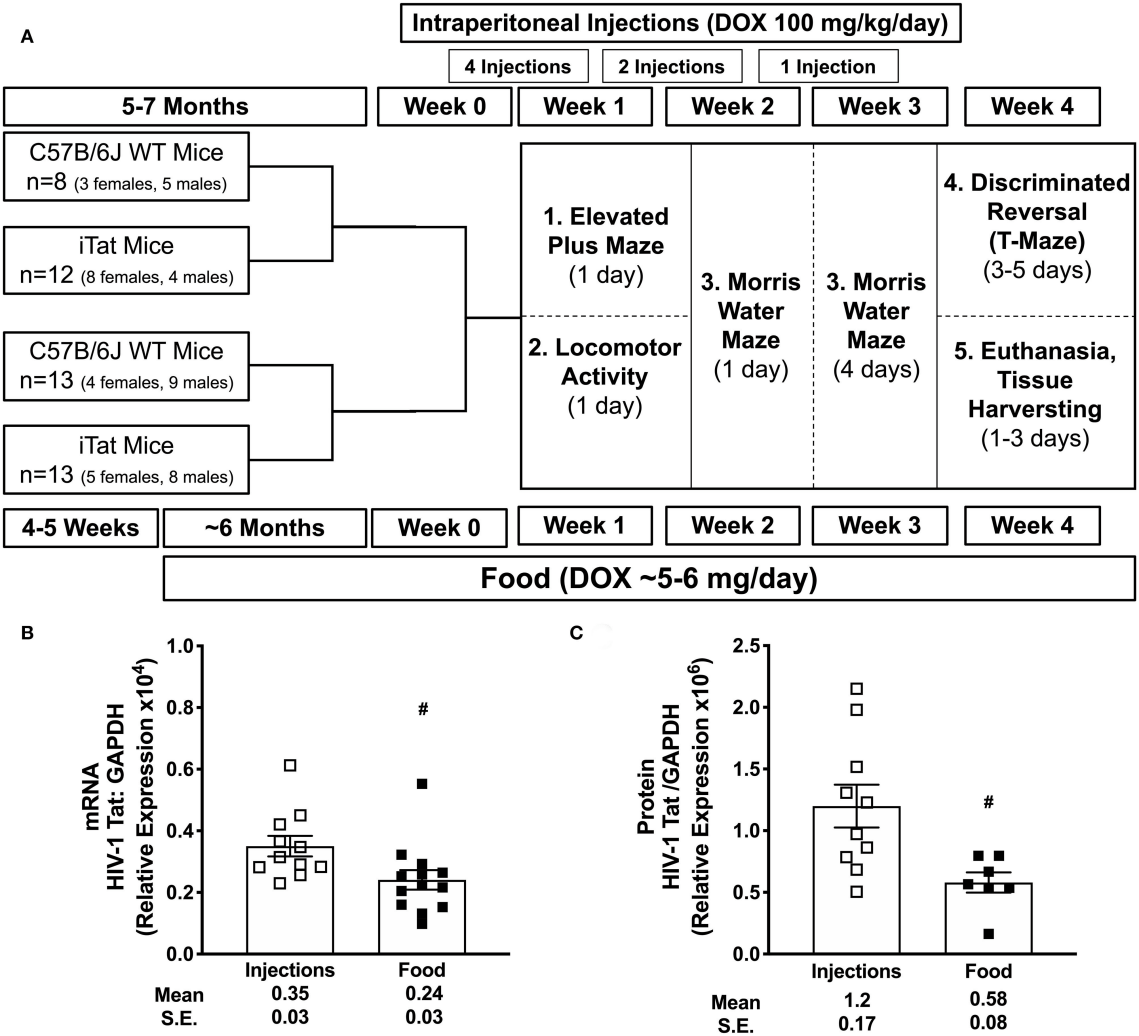
Experimental schematic and acute vs. prolonged *Tat* expression. C57BL/6 wild-type (WT) and iTat mice of both sexes were administered doxycycline (DOX) *via* intraperitoneal (*i.p*.) injections (100 mg/kg/day) or DOX-containing food *ad libitum* (about 5–6 mg DOX/day). **(A)** A total of seven *i.p*. injections were given to 5–7 month old mice over 3 weeks prior to and during behavior testing. In parallel, mice were fed DOX food for about 7 months (6 months prior to and 1 month during the behavior testing) starting at 4 to 5 weeks of age. Therefore, these groups were age-matched at the initiation of behavior testing, which was carried out over 4 weeks. After behavior testing, mice were euthanized, and brain tissues were harvested for gene expression and/or protein analyses. **(B)** Tat mRNA expression was measured in iTat mice using one-step real-time PCR. GAPDH was used as an internal housekeeping control. The iTat mice received acute Tat induction by DOX *i.p*. injections (*n* = 11, open squares) and prolonged Tat induction *via* DOX food *(n* = 13, solid squares). Each bar represents the mean ± SEM. ^#^*p* < 0.050 by unpaired *T*-test. **(C)** Tat protein expression was measured in WT and iTat mice using simple western (WES, protein simple). GAPDH was used as an internal housekeeping control. The WT and iTat mice received acute Tat induction by DOX *i.p*. injections (*n* = 10, open squares) and prolonged Tat induction *via* DOX food *(n* = 7, solid squares). Each bar represents the mean ± SEM. ^#^*p* < 0.050 by unpaired *T*-test.

To evaluate if Tat-mediated effects on MMP/TIMP axis are similar or biphasic in the context of duration of Tat expression, two DOX administration paradigms were compared to induce acute vs. prolonged Tat expression in the iTat mouse model (Figure 1A). First, a series of behavioral tests investigating anxiety, arousal, ambulation, learning, and memory was conducted to validate functional changes characteristic of HAND in these mice. Following behavior testing, brain gene expressions of MMPs, TIMPs and associated proinflammatory mediators were measured.

## Materials and Methods

### Animals

All animal experiments were conducted in strict accordance with the recommendations in the Guide for the Care and Use of Laboratory Animals of the National Institutes of Health. The study and associated protocols were approved by the University of North Texas Health Science Center Institutional Animal Care and Use Committee in Fort Worth, TX prior to initiation of the study. The iTat mice generated as previously described (26) were provided by Dr. Johnny He. iTat mice were rederived by Jackson Laboratory and expressed both transgenes i.e., *hiv tat* and reverse tetracycline-controlled transactivator (rtTA) as confirmed by genotyping. Breeder C57BL6/J wild-type (WT) mice (6 weeks old) were purchased from Jackson Laboratory. All experimental mice were bred in house under the same conditions. The mice were housed in groups of three to five in polycarbonate cages with corncob bedding, fed *ad libitum*, and maintained at an ambient temperature (23 ± 1°C), under a 12 h light/dark cycle.

### Treatments

Wild-type and iTat mice were administered DOX by two different methods, intraperitoneal (*i.p*.) injections or food in order to delineate effects of acute vs. prolonged Tat expression, respectively. We hypothesized that high concentration DOX *via i.p*. injections compared to food (100 mg/kg/day vs. ~5–6 mg/day) would lead to higher and acute Tat expression, whereas low dose DOX *via* food would mimic the low level, mild Tat expression, analogous to chronic phenotype. Age- and sex-matched WT mice were used to evaluate off-target DOX effects. The schematic representation of the experimental timeline is presented in Figure 1A. Additional mice with varied frequencies of DOX injections and food were used in preliminary and/or validation experiments (Supplementary Figure 1A).

#### Acute Tat Induction

Previous studies reported that Tat expression increased significantly after three DOX (100 mg/kg) *i.p*. injections and returned to baseline in 2 weeks after last injection in a similar GFAP promoter-driven HIV-1 Tat expression mouse model (38). To evaluate an optimal injection paradigm in iTat mice, we performed studies with different DOX concentrations, frequency of *i.p*. injections and time course to determine an optimal method to model acute Tat expression. Our initial experimental design included 10 injections over 4 weeks to maintain a high Tat expression over 4 weeks of behavior testing. However, an attrition rate of 35% was observed in iTat mice and 30% in WT mice, potentially due to highly acidic pH of doxycycline hyclate (data not shown). Thus, based on preliminary data and previous reports, seven *i.p*. injections of 100 mg/kg DOX (Cat no. D9891, Sigma-Aldrich, St. Louis, MO) were administered over 3 weeks for an acute Tat induction. Specifically, 5–7 month old mice were injected four times in the week prior to, two times during the 1st week, and once in the 2nd week of behavior testing to maintain Tat expression above baseline during behavioral testing and at the time of brain tissue harvesting.

#### Prolonged Tat Induction

Mice were fed with chow containing DOX (1,250 mg/kg, Cat no. TD.160353, +maltodextrin, green, Harlan Laboratories, Indianapolis, IN) starting at 4 to 5 weeks of age for a total of 6 months prior to and during the 4 weeks of behavior studies to mimic prolonged Tat expression responses. DOX food intake was consistent for 2 months prior to and during behavior studies.

### Behavioral Assessments

At 6 to 7 months, experimental animals were characterized for behavior reflecting anxiety, arousal, spatial learning and memory, and cognitive flexibility. All mice were euthanized within a week after completing behavioral tests. Mice were weighed weekly during behavior studies (Supplementary Figure 2).

#### Elevated Plus Maze (EPM)

To measure anxiety (38), a plus-shaped maze elevated three feet was placed in a dimly lit test room (60 Watts). The maze consisted of two arms opened to the room and two arms enclosed such that the floor and the rest of the room were not visible. An automated tracking system monitored the position of each mouse in the maze (Any-maze, Stoelting Co., Wood Dale, IL). Mice were acclimated to the testing room for a minimum of 10 min prior to testing. Each mouse was placed in the center of the maze facing an open arm and was given 5 min to explore the maze. Percent time spent in the open arms and total distance covered were recorded.

#### Locomotor Activity (LMA)

Spontaneous locomotor activity was measured as described previously (44). In this test, each mouse was placed in a clear acrylic box (40.5 × 40.5 × 30.5 cm), surrounded by a photocells-lined metal frame. The test cage was then placed in a dimly lit chamber equipped with a fan that provided background noise (80 dB). The test was conducted for 16 min, in which movements in the horizontal plane and vertical plane (7.6 cm above the floor of the box) were detected by the photocells and processed by a software program (Digiscan apparatus, Omnitech Electronics, model RXYZCM-16, Columbus, OH) to yield different measures including distance covered, vertical activity, and spatial components of spontaneous activity in the box.

#### Morris Water Maze (MWM)

Spatial learning and memory were measured using a MWM test. Testing was carried out as described previously (45). Mice were acclimated to the testing room for a minimum of 10 min prior to testing each day. During each trial, the mouse was put in a tank filled with opacified water (using non-toxic acrylic white paint) to swim and was able to escape the water by finding and climbing on a platform hidden 1.5 cm below the water surface. The water temperature was maintained at 24 ± 1°C. An automated tracking system recorded various measures such as latency, path length and swimming speed for each trial (Any-maze, Stoelting Co.). The test consisted of two phases. [1] Pre-training phase: during this phase, the tank was covered with a black curtain to hide surrounding visual cues. Each mouse was trained over a single session of five trials with 5 min inter-trial intervals. During each trial, the mouse was allowed to swim until it climbed on the platform or for a maximum of 60 s, whichever was earlier. This pre-training was done so that the mice could learn the motor components of the task such as swimming and climbing onto a platform, and to reduce the bias of anxiety to a new environment during the subsequent phases [2] Acquisition phase: mice were then tested for their ability to locate a hidden platform using spatial cues around the room over four sessions (one session/day). Each session consisted of five trials, at 2-min intervals. For each trial, the mouse was placed at one of four different starting points at the edge of tank and had to swim to the platform, which remained at the same location. The mouse was allowed to swim until it reached the platform or for a maximum of 90 s. Path length (distance taken to reach the platform) over all sessions was used as the primary measure of performance. The swim speed was calculated by dividing path length by the latency (to reach the platform) for each trial.

#### Discrimination Reversal

The discrimination reversal testing assessed memory with a T-maze as described previously (45). Briefly, the T-maze was constructed of acrylic with black sides to hide spatial cues for the mouse and clear tops for the tester to observe the mouse. The maze consisted of three compartments: a start box (10 × 6.3 × 6 cm), which extended into the stem (17.5 × 6.3 × 6 cm), and two arms (14.5 × 6.3 × 6 cm), each separated by acrylic flaps manually operated by the tester. The maze rested on a metal grid wired to deliver 0.69 mA scrambled shock to the feet. Mice were acclimated to the testing room for a minimum of 10 min prior to testing. Each mouse was tested in three sessions separated by 1 h. At the beginning of each trial, the mouse was placed in the start box, and the start flap was removed for the mouse to enter the stem. During the first trial of the first session, the mouse received a mild shock (0.69 mA) on entering an arm (preferred arm) and was allowed to avoid it by running to the other arm, which then became the correct arm for the remainder of first session. For subsequent trials, shock was initiated 5 s after the opening of the start flap if the mouse had not entered the correct arm or immediately upon entry into the incorrect arm. The shock continued until the correct arm was entered or for a maximum of 60 s. Once the mouse entered the correct arm, the flap was closed to prevent escape, and the mouse was moved after 10 s, by detaching the arm, into a holding cage for 1 min before beginning the next trial. This trial paradigm continued until the mouse fulfilled the correct avoidance criterion, i.e., running directly to the correct arm within 5 s, in four of the five consecutive training trials including the last two. In the second and third sessions, there was a reversal in correct arm such that the mouse was required to run to the other arm compared to the one it was trained for in the previous session. The ability of the mice to learn is inversely proportional to number of trials required to fulfill the avoidance criteria.

### Cardiac Perfusion, Euthanasia, and Tissue Harvesting

Mice were euthanized by *i.p*. injections of 100 mg/kg ketamine hydrochloride (100 mg/ml, Putney, Inc, Portland, ME) and 10 mg/kg xylazine (20 mg/ml, Akorn, Inc, Lake Forest, IL) followed by cardiac perfusion using 1X phosphate buffer saline. For animals that underwent behavior testing, each harvested brain was cut in three parts. Posterior half of one hemisphere was used for RNA isolation and subsequent gene expression testing. The posterior half of the other hemisphere was snap frozen prior to protein isolation as described below. The frontal lobe sections were fixed in 4% PFA for future use. Brain tissues of additional mice that received either DOX injections or food at the similar frequencies or duration, respectively, as the mice in the behavior studies were used to elucidate if gene expression patterns observed in different parts of the brain were comparable, namely right hemisphere (RH), left anterior (LA) and left posterior (LP) (Supplementary Figures 1, 3).

### RNA Isolation, cDNA Synthesis, and Real-Time PCR

Tissue was homogenized with Trizol (Sigma Aldrich). The homogenates (1 mL Trizol/~100 mg tissue weight) were centrifuged to remove debris, and viscous supernatants were used for RNA isolation. Total RNA was isolated using phenol-chloroform extraction method, treated with DNAse (Thermo Fisher, Waltham, MA) as per manufacturer's instructions to digest genomic DNA, and then reverse transcribed into cDNA template for real-time PCR. TaqMan™ Fast Advanced Master Mix (Cat no. 4444557, Applied Biosystems, Foster City, CA) and Applied Biosystems TaqMan gene expression assays for TIMP-1 (Cat no. Mm01341361), TIMP-2 (Cat no. Mm00441825), MMP-2 (Cat no. Mm00439498), MMP-9 (Cat no. Mm00442991), interleukin (IL)-1β (Cat no. Mm00434228), tumor necrosis factor (TNF)-α (Cat no. Mm00443258), CCL2 (Cat no. Mm00441242), IL-6 (Cat no. Mm00446190), IL-17 (Cat no. Mm00439618), GFAP (Cat no. Mm01253033), glyceraldehyde 3-phosphate dehydrogenase (GAPDH) (Cat no. 4352339E), beta-actin (Cat no. Mm01205647_g1) and phosphoglycerate kinase (PGK)-1 (Cat no. Mm00435617) were used for gene expression analysis by real-time PCR. Each 20 μL PCR reaction consisted of 100 ng of cDNA, 1X TaqMan™ Fast Advanced Master Mix, 1X probes for both experimental and housekeeping gene targets. The samples were incubated at 95°C for 5 min for polymerase activation, followed by 40 cycles of denaturation and annealing-extension at 95°C for 1 s and 60°C for 20 s. Each sample was measured in triplicates. The threshold cycle (C_T_) values were converted into ΔΔC_T_ to obtain fold-change in expression as compared to treatment WT controls (behavior injection WT *n* = 7, behavior food WT *n* = 13, gross brain region injection WT *n* = 3, gross brain region food WT *n* = 5). We noted a difference in basal GAPDH expression, a commonly used housekeeping control, between both strains (Supplementary Table 1). Subsequently, we tested two other housekeeping genes, *i.e.*, β-actin and PGK-1. PGK-1 was selected to be used as a housekeeping control for gene expression comparisons between strains, based on least difference between the two strains.

For Tat mRNA measurements, mRNA was isolated from total RNA using Dynabeads mRNA direct purification kit (Cat no. 61012, Thermo Fisher). Primers used for Tat detection were 5′ ggaagcatccaggaagtcag 3′ and 5′ ggaggtgggttgctttgata 3′ with 5′ cctcctcaaggcagtcagac 3′ used as probe. Tat mRNA expression was evaluated using TaqMan™ Fast Virus 1-Step Mastermix (Cat no. 4444432, Applied Biosystems). GAPDH was used as an internal housekeeping control. Each 20 μL PCR reaction consisted of 20 ng of mRNA, 1X Fast Virus 1-Step Mastermix, 1X probes for Tat and GAPDH gene targets. The samples were incubated at 50°C for 5 min, 95°C for 20 s, followed by 40 cycles of denaturation and annealing-extension at 95°C for 3 s and 60°C for 30 s. Each sample was measured in triplicates. The ratios of threshold cycle (C_T_) values of Tat and GAPDH were used for quantitative assessments.

### Protein Isolation and Simple Western

Tissue was homogenized in 2 μL RIPA buffer (Thermo Fisher) per mg tissue using a red bead lysis kit with a mix of zirconium beads (Cat no. REDE5, Next Advance, Troy, NY) and the Bead Mill 4 (Fisher) at speed 4 for 3 min. Several WT and iTat brain sections from the food group were homogenized using at 5 μl RIPA/ 1 mg tissue using the PRO200 homogenizer (PRO Scientific, Oxford, CT). However, some of these lysates were too dilute for further analysis. The homogenates were centrifuged for 10 min at 10,000 g to remove the beads and debris. Protein concentrations of the clarified lysates were determined by the BCA protein assay microplate method (Thermo Fisher) according to manufacturer's instructions. Brain lysates were diluted to 2 mg/ml and assayed by simple western (Protein Simple, San Jose, CA) according to their standard protocol. HIV-1 Tat was detected with a rabbit polycolonal antibody (Cat no. ab43014, lot no. GR3264810-9, abcam, Cambridge, MA) at a 1:10 dilution using the 2-40 kDa separation and anti-rabbit detection modules. HIV-1 IIIB Tat recombinant protein was obtained through the NIH AIDS Reagent Program, Division of AIDS, NIAID, NIH and used as a positive control and standard across WES assays. GAPDH was detected using a monocolonal mouse antibody 6C5 (Cat no. sc32233, lot no. K0315, Santa Cruz Biotechnology, Dallas, TX) at a 1:5000 dilution using the 12-230 kDa separation and anti- mouse detection modules. Brain HIV Tat levels were normalized to the HIV-1 Tat positive control column before normalization to GAPDH.

### Statistical Analysis

**Figure 2:**
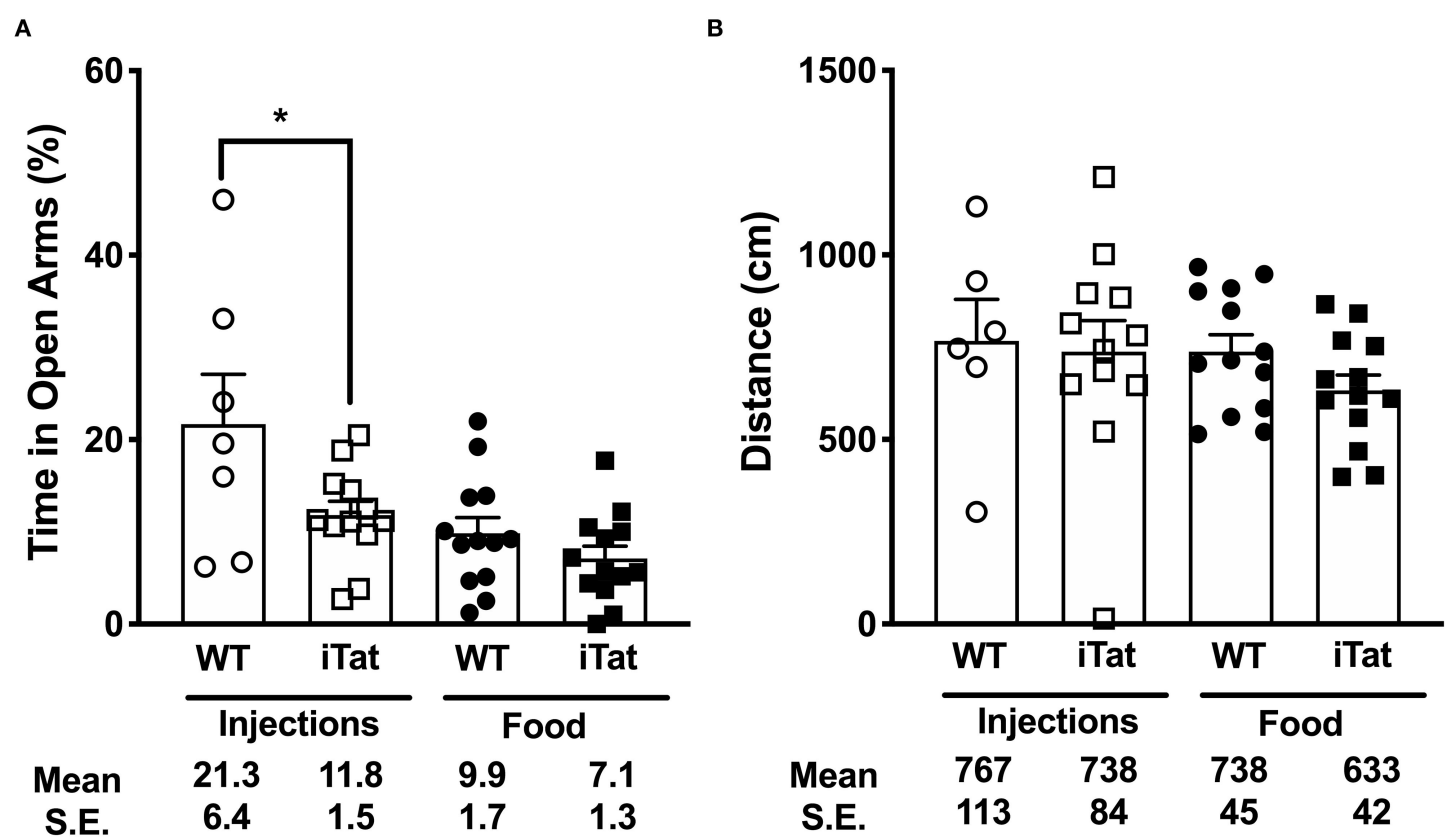
Higher anxiety levels were observed in iTat mice compared to Treatment Time WT controls in elevated plus maze testing. Anxiety levels were measured by **(A)** the percent time spent in the open arms and **(B)** total distance covered on the maze in iTat and WT mice. The WT mice (*n* = 6, open circles) and the iTat mice (*n* = 12, open squares) received acute induction by DOX *i.p*. injections, while the WT mice (*n* = 13, solid circles) and the iTat mice (*n* = 13, solid squares) received prolonged induction *via* DOX food. Each bar represents the mean ± SEM. *p < 0.050 for same Treatment Time comparisons between Strains by two-way ANOVA using Treatment Time and Strain as variables.

**Figure 3:**
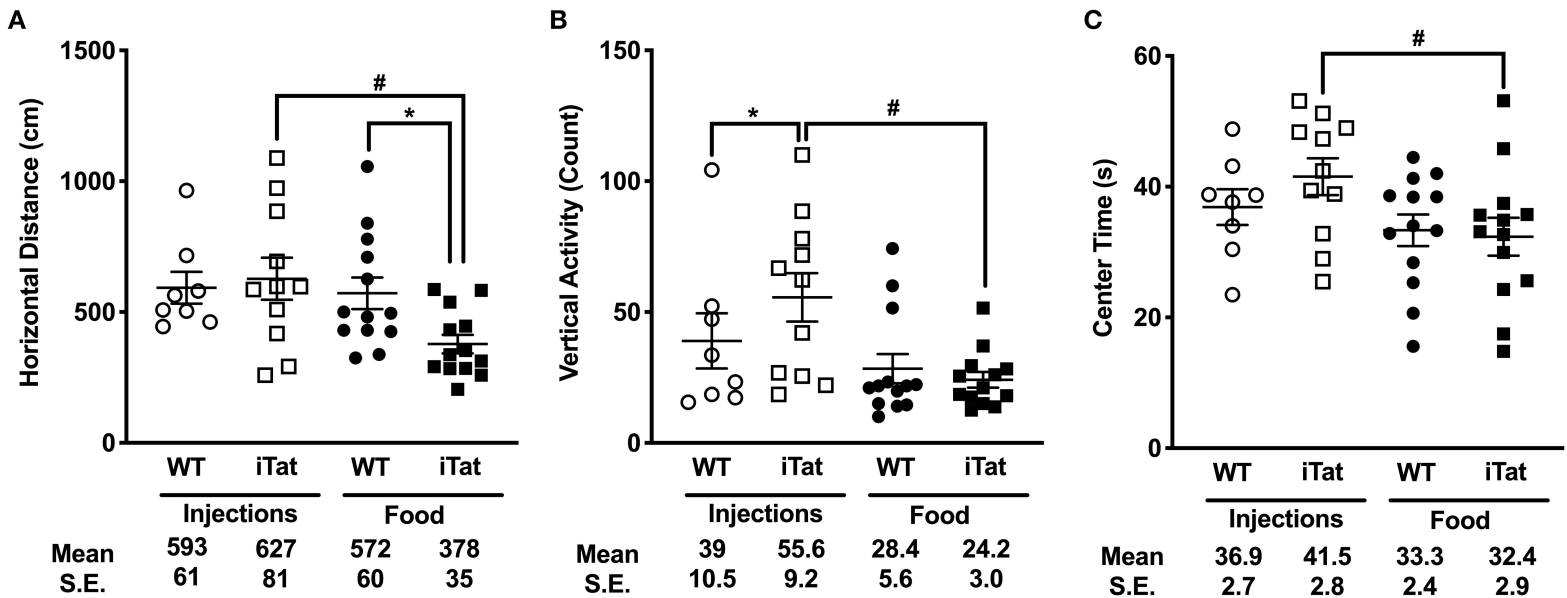
Prolonged HIV-1 Tat expression led to slower ambulation, reduced vertical activity, and reduced center time compared to acute Tat expression in iTat mice. **(A)** Horizontal Distance, **(B)** Vertical Activity, and **(C)** Center Time in iTat and WT mice were measured. Average per minute values for each parameter were plotted. The WT mice (*n* = 7–8, open circles) and the iTat mice (*n* = 10–11, open squares) received acute induction by DOX *i.p*. injections, while the WT mice (*n* = 13, solid circles) and the iTat mice (*n* = 13, solid squares) received prolonged induction *via* DOX food. Each bar represents the mean ± SEM. *p < 0.050 for same Treatment Time comparisons between Strains, ^#^*p* < 0.050 for same Strain comparisons across Treatment Time by two-way ANOVA using Treatment Time and Strain as variables.

**Figure 4:**
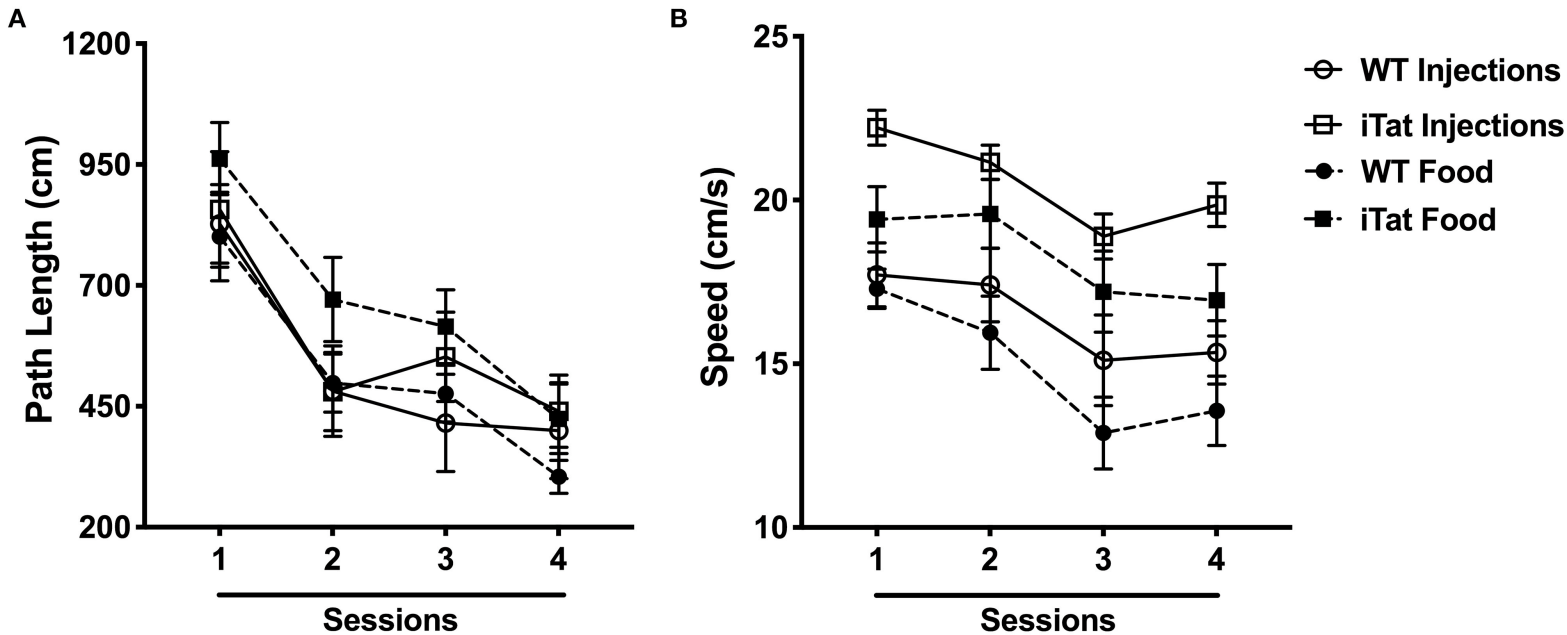
Morris water maze performance indicated faster swim speeds in iTat mice during acute vs. prolonged HIV-1 Tat expression. **(A)** Path length and **(B)** swimming speed were assessed in iTat and WT mice over four sessions. Each data point represents the mean ± SEM from an average of four trials per session. One session is equivalent to 1 day. The WT mice (*n* = 7, solid line with open circles) and the iTat mice (*n* = 11, solid line with open squares) received acute induction by DOX *i.p*. injections, while the WT mice (*n* = 13, dashed line with solid circles) and the iTat mice (*n* = 13, dashed line with solid squares) received prolonged induction *via* DOX food.

**Figure 5:**
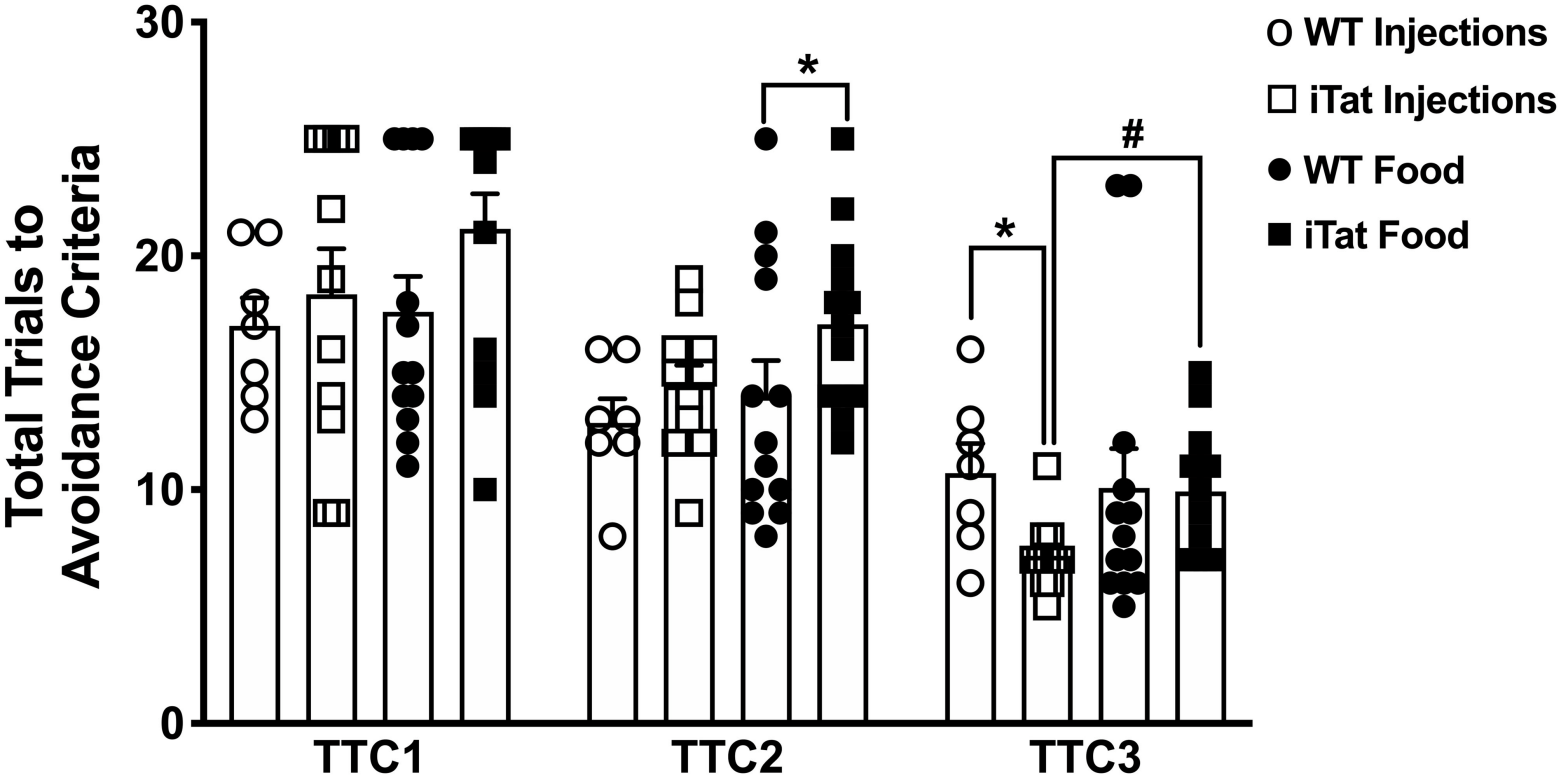
Prolonged HIV-1 Tat expression increased trials to reach avoidance criteria in iTat mice in discrimination reversal test. The T-maze test measured the number of total trials taken by iTat and WT mice to reach discriminated avoidance criteria during three sessions. The WT mice (*n* = 7, open circles) and the iTat mice (*n* = 11, open squares) received acute induction by DOX *i.p*. injections, while the WT mice (*n* = 12, solid circles) and the iTat mice (*n* = 13, solid squares) received prolonged induction *via* DOX food. Each bar represents the mean ± SEM, TTC: total trials to criteria, TTC1: Acquisition session, TTC2, TTC3: Reversal session, *p < 0.050 for same Treatment Time comparisons between Strains, ^#^*p* < 0.050 for same Strain comparisons across Treatment Time by two-way ANOVA using Treatment Time and Strain as variables.

**Figure 6:**
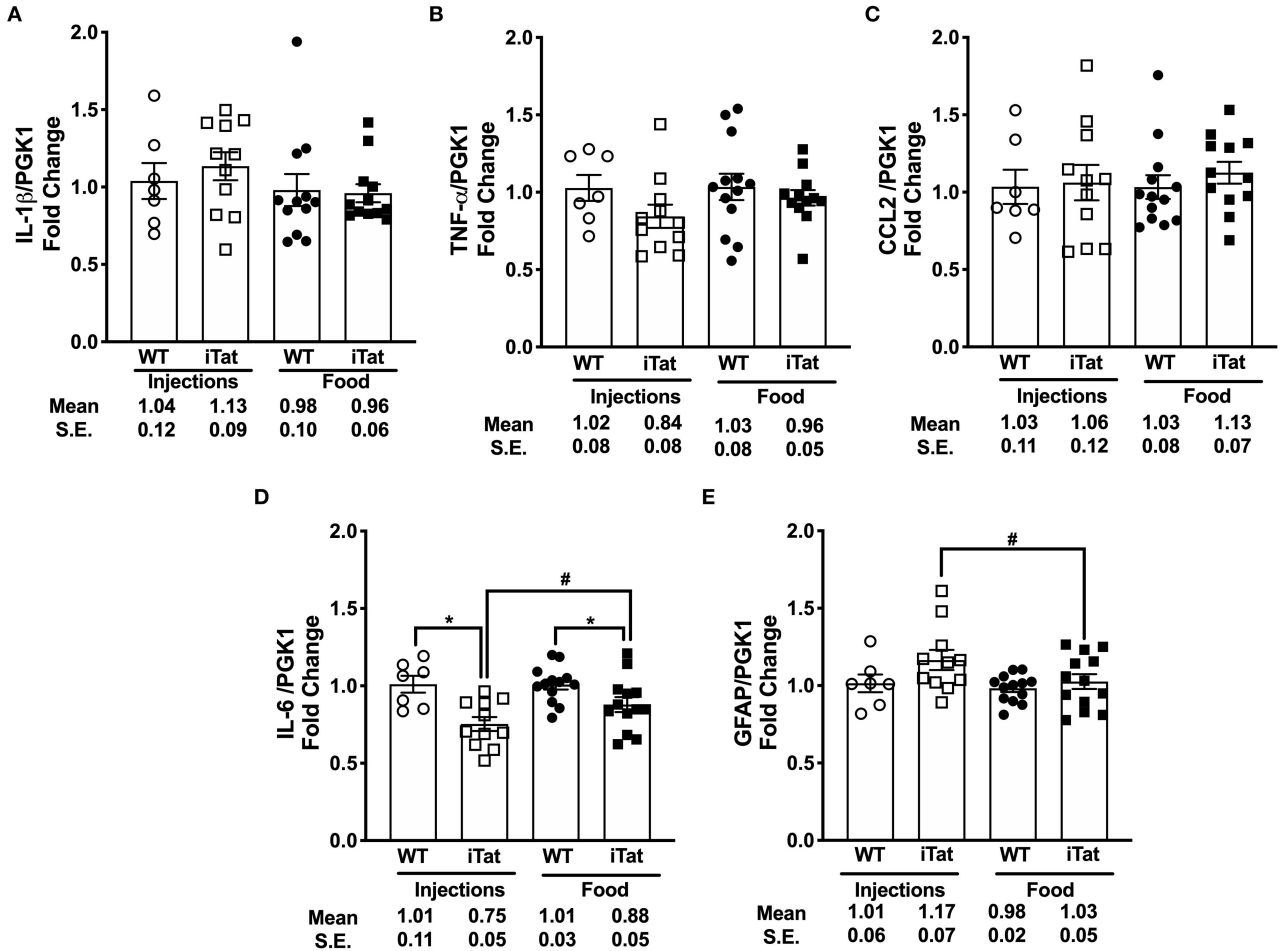
Key pro-inflammatory biomarkers gene expression remained comparable with acute and prolonged HIV-1 Tat induction in iTat mice. **(A)** IL-1β **(B)** TNF-α **(C)** CCL2, **(D)** IL-6, and **(E)** GFAP mRNA levels were measured in iTat and WT mouse brain tissues by real-time PCR. The WT mice (*n* = 7, open circles) and the iTat mice (*n* = 11, open squares) received acute induction by DOX *i.p*. injections, while the WT mice (*n* = 12–13, solid circles) and the iTat mice (*n* = 12–13, solid squares) received prolonged induction *via* DOX food. Each bar represents the mean ± SEM, *p < 0.050 for same Treatment Time comparisons between Strains, ^#^*p* < 0.050 for same Strain comparisons across Treatment Time by two-way ANOVA using Treatment Time and Strain as variables.

**Figure 7:**
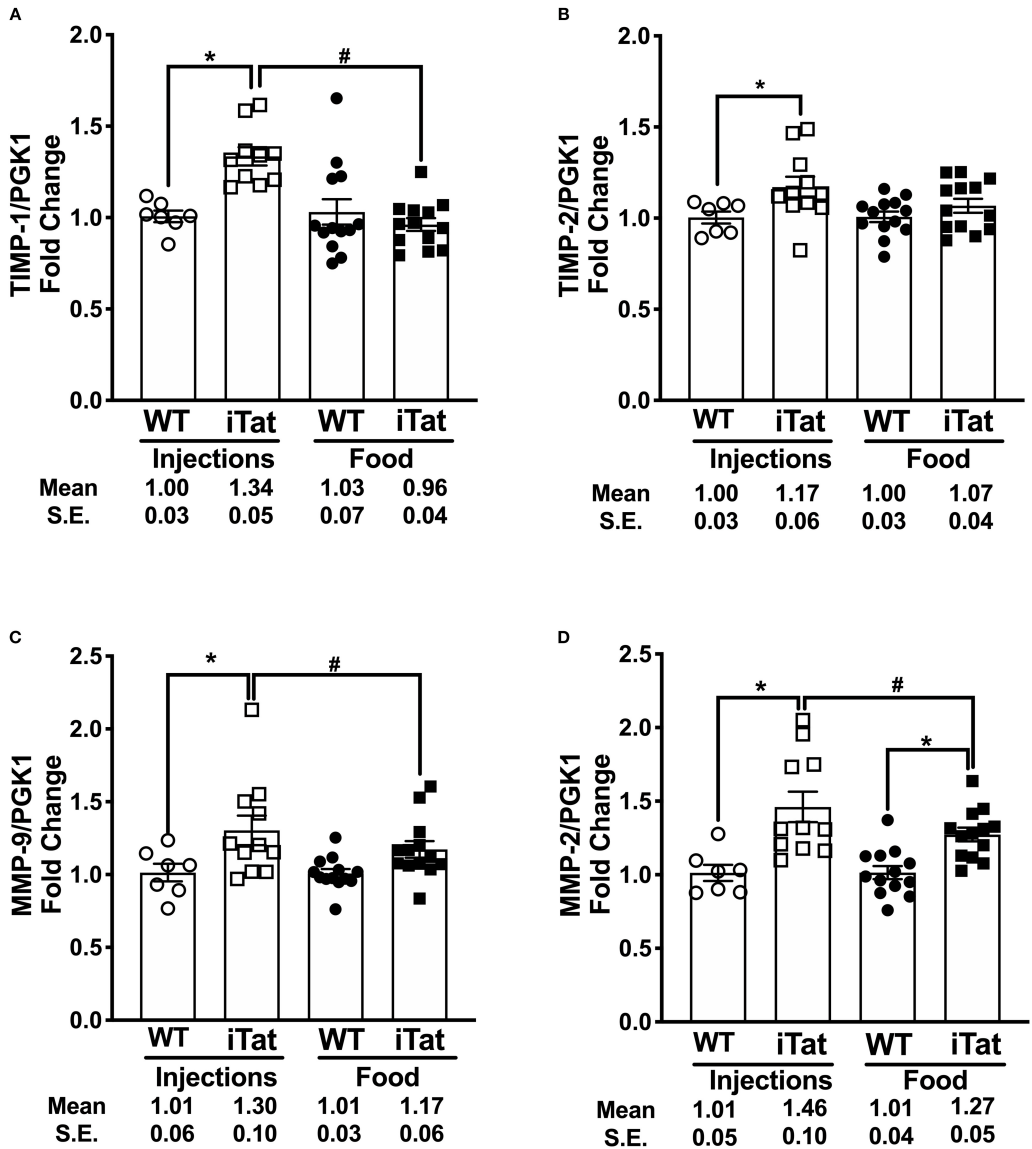
Acute vs. prolonged HIV-1 Tat induction dysregulated MMP/TIMP balance in iTat mice. **(A)** TIMP-1, **(B)** TIMP-2, **(C)** MMP-9, **(D)** and MMP-2 gene expression was measured in iTat and WT mice. The WT mice (*n* = 7, open circles) and the iTat mice (*n* = 11, open squares) received acute induction by DOX *i.p*. injections, while the WT mice (*n* = 12–13, solid circles) and the iTat mice (*n* = 12 – 13, solid squares) received prolonged induction *via* DOX food. Each bar represents the mean ± SEM, *p < 0.050 same Treatment Time comparisons between Strains, ^#^*p* < 0.050 for the same Strain comparisons across the treatments by two-way ANOVA using Treatment Time and Strain as variables.

All data are presented as mean ± SEM. Differences between mean values were determined using analyses of variance (ANOVA) or *T*-test. Tat mRNA and protein levels in iTat mice between two induction paradigms were analyzed using a two-sided *T*-test. All behavior and gene expression data were subjected to two-way ANOVA, with Treatment Time (acute vs. prolonged) and Strain (WT vs. iTat) as between group factors. Additionally, Water maze and body weight data were subjected to three-way ANOVA, with Treatment Time and Strain as between-group factors, and duration or session as within group factors. Planned individual comparisons between groups were made using the single degree-of-freedom F tests. The alpha level was set at 0.05 for all analyses. Statistical analyses for behavioral tests and gene expression post behavior testing were performed using Systat 13 (Figures 2–**7**). Gene expression data for Tat mRNA, protein and brain region-specific gene expression in additional mice were analyzed using Prism 8.0 (Figure 1, Supplementary Figures 1, 3).

## Results

### Evaluating Tat Transgene Expression

Acute Tat expression was induced using DOX injection method, similar to previous studies investigating Tat-mediated behavioral changes in iTat mice (36, 38, 39). Additionally, prolonged Tat expression was induced *via* food, which was also used in this model previously (43). Tat gene expression was quantified in mRNA isolated from iTat mouse brain tissues using one-step PCR. Preliminary assessments for acute induction model indicated that, Tat expression increased significantly with increased number of DOX injections increased from six injections to ten injections and reduced sharply after a week while remaining at detectable levels (Supplementary Figure 1B). Further, Tat expression was higher in the left posterior (LP) brain region (*p* = 0.013 and *p* = 0.023, respectively), compared to right hemisphere (RH) and left anterior (LA) with the *i.p*. induction method, while prolonged food-based method did not show changes in Tat expression in different gross brain regions (Supplementary Figure 1C). In the mice that underwent behavior studies, relative *tat* gene expression was evaluated in brain harvested at the end of the study. Tat mRMA levels were higher in acute iTat brains (injection) compared to prolonged iTat brains (Figure 1B) (^#^*p* = 0.026). Tat gene expression was undetectable in WT brain tissues (data not shown). Similar to mRNA levels, Tat protein levels were significantly higher than DOX-injected iTat mice compared to DOX-fed mice (^*^p = 0.013) (Figure 1C).

### Elevated Plus Maze (EPM)

Anxiety was measured using the percent time spent in the open arms of the EPM (Figure 2A). In the injected group, the iTat mice spent less time than their WT controls in the open arms (*p* < 0.05), while in the food group, there was no significant difference between the iTat mice and their WT controls. Overall, the DOX fed mice spent less time in the open arms than the DOX injected mice, and the iTat mice, regardless of treatment, spent less time in the open arms than the WT. These observations were supported by an ANOVA, which revealed significant main effects of Treatment Time (*p* = 0.002) and Strain (*p* = 0.014) on the percent time spent in the open arms; however, there was no significant interaction between the two factors (*p* = 0.170).

Distance traveled by the mice in the maze was measured to determine if activity affected the time spent in the arms (Figure 2B). There was no difference between any of the groups, which was supported by a lack of significant main effects of Treatment Time (*p* = 0.342), Strain (*p* = 0.339), or their interaction (*p* = 0.586).

### Locomotor Activity (LMA)

The effects of Treatment Time and Strain on the horizontal, vertical, and spatial components of spontaneous activity are presented in Figure 3. In the injected group, there was no difference between the iTat and the WT, while in the food group, the iTat traveled less distance than the WT (*p* < 0.050) (Figure 3A). The two-way ANOVA revealed a significant main effect of Treatment Time (*p* = 0.034), but no effect of Strain (*p* = 0.202) or an interaction between Strain and Treatment Time (*p* = 0.071).

The number of rearing counts served as a measure for vertical activity and is presented in Figure 3B. In the injection group, the iTat mice had higher rearing counts compared to their WT (*p* < 0.050), while in the food group, there was no difference between iTat and WT. This finding was supported by a significant interaction of Treatment Time and Strain (*p* = 0.040). The two-way ANOVA also yielded a significant main effect of Treatment Time (*p* = 0.030) but no effect of Strain (*p* = 0.180).

In Figure 3C, the amount of time spent in the center of the apparatus is presented. In both injected and food groups, the time spent in the center was comparable between WT and iTat. Overall, there was no difference between the WT and iTat mice; however, the injected mice spent more time in the center than the food mice. These observations are supported by a two-way ANOVA yielding a main effect of Treatment Time (*p* = 0.029), and no effect of Strain (*p* = 0.516) or interaction of the two factors (*p* = 0.322).

Given the differences observed in acute vs. prolonged iTat mice, we further evaluated if these were driven by Sex as a secondary analysis. However, in a three-way ANOVA using on all measures of LMA there was no significant interaction of Sex with Strain or Treatment. We also looked at averages for each and similar trends were seen in both sexes supporting the lack of interaction reported by the analyses.

### Morris Water Maze (MWM)

Spatial memory was assessed measuring path length and swim speed of mice to locate a hidden platform under the water surface (Figure 4A). All mice learned to locate the platform across sessions, and a similar pattern of learning efficiency was observed across the groups. This was supported by a significant effect of Session (*p* < 0.05) and a lack of interaction between Session and any other factors (all *p* > 0.400) following a repeated measure ANOVA. In the injected group, there was no difference between iTat and WT, while in the food group, the iTat took longer path length at every session than the WT. This observation was not supported by a main effect of Strain (*p* = 0.089) or Treatment Time (*p* = 0.516), or an interaction between the two factors (*p* = 0.399).

Swim speed was analyzed and is presented in Figure 4B. The swim speed of the mice varied across sessions (*p* < 0.010), but the other factors did not have an effect across sessions (all *p* > 0.2). Overall, the iTat mice swam faster than the WT mice in both paradigms, and the injected mice seemed to swim faster than the food mice, but it was mostly due to the injected iTat mice. These observations were supported by a main effect of Strain (*p* < 0.001), a main effect of Treatment Time (*p* = 0.049), and no interaction between two factors (*p* = 0.676).

### Discrimination Reversal (T-Maze)

Data from the first session of the discriminated avoidance task (TTC1) represent a measure of learning/acquisition and the subsequent two sessions represent a measure of cognitive flexibility (TTC2, TTC3) (Figure 5). During the acquisition session, even though the food iTat mice seem to take more trials to reach criterion it did not reach significance. Neither strain nor treatment time seem to affect the performance of the mice during acquisition, which is supported by lack of main effects or interaction following a two-way ANOVA (all *p* > 0.132) (Figure 5). During the first reversal session (TTC2), there was no difference between the two genotypes in the injected group. However, in the food group, the iTat mice took more trials to reach criterion compared to the WT (*p* < 0.05). A two-way ANOVA yielded a main effect of Strain (*p* = 0.021) which was mostly due to the effect in the food group, and there was no main effect of Treatment Time or an interaction (all *p* > 0.226). In the last reversal session (TTC3), the injected iTat mice took less trials than the WT, while the food iTat mice took more trials than the WT to reach criterion. This observation was supported by a significant interaction between Strain and Treatment Time (*p* = 0.041).

### Evaluating Expression of Proinflammatory Cytokines and Gliosis

The mRNA levels of a number of proinflammatory cytokines including IL-1β, TNF-α, and CCL2 were comparable among all treatment groups (Figures 6A–C). Subsequently, a two-way ANOVA failed to show main effects of Treatment Time and Strain or their interaction on their gene expression (all *p* > 0.305). The gene expression of IL-6 was significantly reduced in iTat mice compared to Treatment Time WT controls, respectively (both *p* < 0.050) (Figure 6D). Further, the IL-6 expression was significantly higher in DOX-fed iTat mice (*p* = 0.044) compared to DOX-injected iTat mice. A two-way ANOVA indicated a main effect of Strain (*p* < 0.001), but there was no effect of Treatment Time or interaction of the two-factors. Gene expression of another known inflammatory cytokine IL-17 was undetectable in both WT and iTat mice (data not shown). As the Tat transgene expression is driven by the GFAP promoter and elevated GFAP expression is a marker of astrogliosis, we measured if there were transcriptional changes in GFAP expression. While GFAP mRNA expression in acute and prolonged Tat expressing mice was comparable to their respective WT controls, acute Tat expressing mice showed significantly higher GFAP mRNA expression when compared to prolonged Tat-expressing mice (*p* < 0.05) (Figure 6E). This was also reflected in a main effect of Strain approaching significance (*p* = 0.062); however, there was no effect of Treatment Time (*p* = 0.097) and its interaction with Strain (*p* = 0.298) on GFAP gene expression.

### Gene Expression of MMP/TIMP Balance Components

Simultaneously, we evaluated the MMP/TIMP balance in iTat mice, by measuring TIMP-1, TIMP-2, MMP-9, and MMP-2 gene expression profiles (Figure 7). Among inducible proteins regulating the MMP/TIMP axis, TIMP-1 mRNA levels increased significantly in acute Tat-expressing mice compared to their WT controls, while prolonged Tat expressing iTat mice showed comparable expression to their respective WT mice (Figure 7A). More importantly, TIMP-1 expression was significantly lower in prolonged Tat-expressing iTat mice compared to their acute Tat-expressing counterparts. These trends were reflected in the two-way ANOVA, which showed main effects of Treatment Time (*p* = 0.015) and Strain (*p* = 0.043) as well as their interaction on TIMP-1 gene expression (*p* = 0.009). Acute Tat-expressing iTat mice had higher TIMP-2 mRNA expression compared to their WT control (Figure 7B). The levels of MMP-9 and MMP-2 were higher in iTat mice compared to their WT (Figures 7C,D). A two-way ANOVA indicated a main effect of Strain (all *p* < 0.011) on TIMP-2, MMP-9, and MMP-2 levels; however, there were no effects of Treatment Time and its interaction with Strain (all *p* > 0.134). The changes in mRNA expression were also analyzed as MMP/TIMP ratios for iTat mice (Table 1). Both MMP-2 and MMP-9 ratios to TIMP-1 were higher in the prolonged Tat-induction paradigm compared to acute Tat-expressing iTat mice. On the other hand, MMP-2 ratio to TIMP-2 was comparable across Treatment Time for iTat mice. Our preliminary studies also established that relative changes in the mRNA expression of MMP/TIMP components remained consistent in different parts of the brain (Supplementary Figure 3) and changes observed following behavior studies were consistent in mice that did not undergo behavior, highlighting that there was no effect of behavior studies and/or gross brain regions on the changes reported here.

**Table 1:** MMP/TIMP ratios in acute and prolonged DOX-treated iTat mice injected and fed with DOX, respectively.

**Ratio**	**iTat Inj**	**iTat Food**
	***n* = 11**	***n* = 13**
MMP-9/TIMP-1	0.95 (0.38)	1.24 (0.3)^#^
MMP-2/TIMP-2	1.26 (0.28)	1.20 (0.2)
MMP-2/TIMP-1	1.05 (0.38)	1.34 (0.25)^#^
MMP-9/TIMP-2	1.13 (0.32)	1.11 (0.21)

TIMP-1, TIMP-2, MMP-9, and MMP-2 mRNA fold change values presented in Figure 7 were used to obtain the above ratios in iTat mice. The ratios were calculated from the mean of relative fold change calculated for iTat mice, relative to their treatment time matched WT controls. Each row represents the mean ± Standard Deviation

# *p < 0.05 for same Strain comparisons across Treatment Time by one-way ANOVA*.

## Discussion

The iTat mice used in this study (26, 46) and a similar inducible HIV-1 Tat model (35, 47) have been used to evaluate behavior (36, 38, 43, 48) and/or gene expression (49, 50) following either DOX *i.p*. injections or food. In this study, both DOX-administration methods, *i.e., i.p*. injections and food, were used simultaneously to mimic acute vs. prolonged Tat induction, respectively. A previous study concurrently evaluated acute and prolonged effects of Tat in Sprague-Dawley rats; however, resulting changes from both methods were not directly compared (31). Further, induction methods consisted of direct Tat injection in the brain for acute effects, while chronic expression was achieved *via* injection of SV40-derived vector expressing Tat (31). Thus, to the best of our knowledge, this is the first report directly comparing two different HIV-1 Tat induction methods to evaluate changes in behavior and gene expression in a rodent model.

We showed that Tat induction in iTat mice led to mild behavioral deficits when compared to WT controls including one or more of the following trends [1] higher anxiety levels depicted by reduced time spent in the open arms of EPM [2] altered ambulation during LMA [3] higher swim speeds in MWM [4] altered learning in T-maze. Simultaneously, we also depicted that gene expressions of [1] select inflammatory cytokines were unchanged, [2] MMPs were elevated, [3] TIMPs were altered depending on the duration of Tat exposure, subsequently impacting the brain MMP/TIMP balance. Lastly, when Treatment Time was considered as a variable, DOX-injected iTat mice were distinctly different as compared to DOX-fed iTat mice in terms of behavior and gene expression.

Psychiatric conditions including anxiety disorders are frequently observed in PLWH (51). HIV-1 proteins including Tat and glycoprotein120 (gp120) are implicated in direct or indirect mechanisms that manifest into anxiety-like symptoms (52). Indeed, several previous reports investigated Tat-induced anxiety and/or stress, including sex-specific differences, in transgenic HIV-1 Tat rodent models (42, 43, 48, 53). Since our investigations did not focus on sex-specific changes, we did not test for sex as a confounding variable for anxiety. Paris et al. reported a DOX *i.p*. dose- and duration-dependent increase in anxiety using open field and marble burying tests in iTat mice (38). The data from our studies is consistent in part with these findings, as we observed increased anxiety in EPM in DOX-injected iTat mice compared to their WT counterparts. However, such difference in anxiety was not observed in DOX-fed iTat mice, despite a much longer duration of Tat expression. Reduced ambulation is also among metrics for increased anxiety (54). Additionally, increased swim speed in MWM was associated with higher stress in mice (55) as well as rats (56). Thus, data from this study for DOX-injected vs. DOX-fed iTat mice, as compared to their Treatment Time WT controls, collectively present two overlapping, yet distinct behavioral phenotypes; related to higher anxiety and stress. The DOX-injected mice spent less time in the open arms of the EPM and swam faster in MWM but showed no differences in spontaneous locomotor activity. On the other hand, DOX-fed mice displayed lower ambulation in LMA and swam faster in MWM; yet, showed no differences in EPM. In turn, these findings indicate that there are multiple underlying mechanisms that modulate the anxiety-like behaviors, which are potentially regulated by varied Tat concentrations and/or exposure durations.

Impaired motor skills, characterized by slowed movements and incoordination, were among the symptoms of the mild cognitive motor disorder (MCMD) as per the 1991 American Academy of Neurology criteria (57). In the revised HAND classification system, commonly known as Frascati criteria, motor skills testing was included in the cognitive neuropsychological assessments (4). Further, motor symptoms such as leg weakness and unsteady gait were observed in mild forms of HAND (4). In this context, the lowered ambulation observed in DOX-fed mice was consistent with previous reports measuring ambulation using same test and model (43) as well as a different HIV-1 Tat model, which measured ambulation using open field test (48). In parallel, the DOX-injected iTat mice showed similar ambulation, center time, and higher vertical activity as compared to their Treatment Time controls. Thus, we observed Treatment Time-specific difference in iTat mice for all three measures of LMA. It might be argued that these differences were impacted by body weight since the DOX-fed mice had an average 33% higher body weight. However, such distinction was not observed in DOX-injected and -fed WT mice, despite exhibiting a similar weight difference. Thus, it can be inferred that the differential Tat exposure led to distinct locomotor changes in iTat mice.

Lastly, we used MWM and T-maze to study spatial learning and memory, and cognitive flexibility. We did not observe robust changes between iTat and WT mice by either induction paradigm, except the DOX-fed iTat mice demonstrated poorer learning during the second reversal trial in T-maze. These data were in contrast to previous literature that found distinct memory and cognitive impairments (36, 43, 58, 59). It is possible that differences observed in behavioral trends in this study compared to previous reports could be attributed to different readouts in a test, such as MWM conducted over 4 days in this study vs. 18 days in a previous study (43), or due to differences in two strains of Tat mice, which differ in Tat copy number (42). Additionally, two induction paradigms present different handling requirements and it is not known if additional handling of mice during injection may change their response to behavior.

Our approach to mimic acute Tat expression using *i.p*. injections was based previous reports that depicted dynamic dose- and frequency-dependent changes in Tat expression (36, 38). In contrast, studies employing DOX-containing chow ranging from 3 weeks to 1 year have emphasized on the paradigm mimicking a chronic, low level inflammation, and leading to detrimental effects including reduced brain volume, increased ventricular volume, gliosis, inflammatory cytokine expression, and neuronal damage (42, 43, 48, 50). In this regard, our study provides a first direct comparison of Tat expression by two DOX administration methods. As stated earlier, the objective for evaluating behavior prior to studying MMP/TIMP axis in these mice was to evaluate if the behavioral changes are consistent in both induction paradigms and if the observed phenotype would be analogous to symptoms observed in HAND patients. Both paradigms resulted in changes including increased anxiety, motor and/or learning deficits. However, higher Tat levels resulting from DOX *i.p*. injections did not translate into relatively increased deficits when compared to mildly elevated Tat expression *via* DOX-containing chow. Further, the high expression obtained with DOX-injections reduced rapidly and there was a high attrition rate observed. Alternately, the food-based induction provided a mild, yet sustained expression over a longer time with minimal to no attrition. These observations highlight that Tat induction method should be selected carefully depending on the goals of the study.

Previous reports documented that Tat-induced behavioral impairments were sex dependent and males were more vulnerable than female mice (48, 60). Further, direct intervention with progesterone reduced anxiety-like effects in ovariectomized female iTat mice indicating direct effect of sex hormones in modulating Tat-induced behavioral impairments (53). In this context, we performed a preliminary analysis of our data to evaluate sex-based differences in behavior. It was observed that characteristic behavioral deficit phenotype may be more prominent in male mice compared to female mice. However, further testing with more mice and thorough analysis will be required to evaluate these trends further.

Despite milder symptoms observed in PLWH during the ART era, underlying neuropathogenesis remains persistent and complex involving multiple underlying processes such as gliosis, elevated inflammatory factors, and neurotoxicity (6). Alterations in the tightly regulated brain MMP/TIMP balance is among the phenomena associated with neuroinflammation and intensively studied in multiple neurological conditions (8, 61). The principal MMP-driven mechanisms contributing to HAND pathogenesis include blood-brain barrier (BBB) breakdown, inducing neuronal dysfunction, and myelin degradation (11). The effects of MMPs are inhibited by TIMPs, mainly TIMP-1 and TIMP-2. TIMP-1 is produced in response to injury by multiple cell types including reactive astrocytes (62) and reactive astrogliosis is one of the hallmarks of chronic neuroinflammation characteristic of HAND (63). Therefore, it is essential to understand brain TIMP-1 regulation in the HAND-relevant iTat model.

It must be noted that the iTat mouse model produces only one viral protein, *i.e.*, HIV-1 Tat, in a specific cell type, *i.e.*, astrocytes. Astrocytes are highly relevant in HAND pathology, as they are infected by HIV, possess an ability to harbor virus, and remain a latent viral reservoir in the brain (64–66). Astrocytes are also capable of producing viral proteins such as Nef and Tat (67, 68). Transcriptomic analysis in an *in vitro* model of latently infected human astrocytes found upregulated neuroinflammatory pathways including interferon signaling, death receptor signaling, and activation of pattern recognition receptors (69). Another recent study demonstrated that astrocyte-derived HIV trafficked out of CNS and was found in peripheral organs in a transgenic mouse model (3). Thus, while it is not possible to mimic HAND in its entirety in iTat mice, it remains a clinically relevant model to understand impact of astrocytes on neuroinflammation in HAND.

We and several others depicted inflammatory biomarkers TNF-α- or IL-1β-mediated MMP and/or TIMP expression regulation *in vitro* or *in vivo*, particularly in astrocytes (24, 70–72). Further, Tat-mediated upregulation of TNF-α was linked to MMP regulation and subsequent neurotoxicity (33, 73). In a study that found altered MMPs and TIMPs levels in the CSF and blood of HAND patients, TNF-α and CCL2 were among top three altered proinflammatory cytokines (23). A recent study in a similar Tat-transgenic model demonstrated increase in TNF-α, IL-1β, CCL2, IL-6, and IL-17 gene expression in the cortex with prolonged Tat expression (50). Contrary to these findings, the basal levels of IL-1β, TNF-α, CCL2 were comparable to respective WT controls in our studies. In parallel, IL-6 mRNA expression was reduced in iTat mice compared to WT mice and IL-17 was not detected in the brains of both WT and iTat mice. Our results suggest that Tat expression may not directly upregulate these select inflammatory cytokines transcriptionally in this model. It remains to be seen if the baseline expression levels of these cytokines regulate MMPs and TIMPs transcriptionally in addition to Tat-driven mechanisms. Simultaneously, it is also possible that using PGK-1 as a housekeeping gene instead of commonly used GAPDH may alter the gene expression results and the presented data must be interpreted accordingly.

Lastly, we observed marginal increases in GFAP transcription in iTat mice that were not significant compared to WT controls. However, GFAP gene expression in DOX-injected iTat mice was higher than DOX-fed mice. Dr. He and colleagues demonstrated a Tat-induced GFAP protein elevation in iTat mice in several reports (46, 49). Our data corroborate this on a transcriptional level since changes in GFAP in acute vs. prolonged Tat expression correspond with relative Tat levels in both paradigms.

All four tested MMPs and TIMPs were elevated in DOX-injected iTat mice, which showed a higher Tat expression. In parallel, DOX-fed iTat mice, with lower Tat expression, indicated comparable levels of inducible proteins TIMP-1, MMP-9, as well as, TIMP-2. These results suggest that Tat-induced transcriptional changes in MMP/TIMP axis might be driven by reversible, negative feedback mechanisms. This hypothesis is also supported by the results observed in our preliminary experiments. In the preliminary experiments, mice euthanized 1 day after six DOX injections showed robust changes in MMP and TIMP gene expression. In comparison, increases in gene expression of MMPs and TIMPs were not as robust in DOX-injected mice that underwent behavior and were harvested 2 weeks after the last DOX injection. Further, both TIMP-1 and TIMP-2 have known neurotropic and neuroprotective effects (74, 75), and their return normal levels during prolonged inflammation tipped the scale toward MMPs.

These results validated our hypotheses that TIMP-1 is a key regulator in maintaining the brain MMP/TIMP balance and it is differentially regulated during acute vs. prolonged inflammatory stimuli. It was documented that TIMP-1 and TIMP-2 preferentially inhibit MMP-9 and MMP-2, respectively (74). In this context, the MMP-9/TIMP-1 ratio was investigated extensively in multiple neurological conditions such as multiple sclerosis (76, 77), stroke/ischemia (78, 79), and Alzheimer's disease (80). Our data confirms that both of these ratios are relevant in the context of HAND as MMP-2/TIMP-2 ratio remains higher in iTat mice compared to WT mice irrespective of the duration of Tat exposure whereas elevated MMP-9/TIMP-1 may be used to predict prolonged inflammation in this model. There are a couple of potential caveats that should be considered in the context of presented data. First, previous reports documented DOX induced transcriptional inhibition of MMPs (81) and hence using a DOX-inducible transgenic model for studying gene expression of MMP/TIMP axis may not be appropriate. However, we used appropriate WT controls that were either injected or fed with DOX, potentially eliminating DOX-specific effects from interpretation. Further, despite high amounts of DOX administration, significant changes were observed in MMPs. Second, our study does not indicate a direct Tat-mediated regulation of MMP/TIMP balance and further investigations will be required to delineate specific underlying mechanisms. Overall, our data emphasizes the potential role of MMP/TIMP axis during Tat-mediated neurotoxicity.

A previous report documented that CSF MMP-9 and MMP-2 had deleterious effects on verbal fluency and motor speed parameters, respectively (82). More recently, we reported that plasma levels of TIMP-1 correlated significantly with neurocognitive performance measures including complex attention, cognitive flexibility, psychomotor speed, and executive function in HIV+ cohort. In this regard, it is logical to speculate the impact of MMP/TIMP axis on behavioral changes observed in iTat mice. This hypothesis is supported by previous studies, which found impact of MMP/TIMP axis components on rodent behavior. In particular, TIMP-1 was shown to impact memory and cognition in mice in an olfactory maze test (83, 84) and MMP-9 knockout mice exhibited lower anxiety in EPM and higher vertical activity in an open field test (85). The MMP-9/TIMP-1 balance was also associated with altered neuronal plasticity and memory in rodent models (86, 87). Further, increased MMP levels are also linked to long-term memory (85, 87) as well as learning impairments (83, 84), and altered synaptic plasticity (86). Since the link between dysregulated intracellular mechanisms and behavioral phenotypes in HAND is not yet well-established, we infer that our data provides a potential novel direction for HAND investigations.

## Conclusion

As per recent reports, neurological complications in PLWH have become less severe with impairments related to working memory, executive functioning, and speed of information processing (88). The mild behavioral impairments observed in our studies provide a relevant model for future tests on the effects of treatment and/or mitigating interventions. The gene expression established that prolonged Tat expression tipped the MMP/TIMP axis toward MMPs in iTat mice. Consistent with our previous work, TIMP-1 expression increased with acute Tat expression and was reduced in comparison during prolonged Tat expression (24, 25). Considering the MMP-independent neuroprotective functions of TIMP-1 (14, 15, 17, 89), these findings support our hypothesis of TIMP-1 restoration as a therapeutic strategy to treat or prevent neurological deficits in HAND. Astrocytes are the primary producers of TIMP-1 in the brain following injury or inflammatory stimuli. Thus, a tested, efficient, astrocyte-targeted gene therapy approach (90, 91) to modulate TIMP-1 would be the most logical next step. These future studies could have broader implications not just for HAND, but other neurodegenerative conditions as well.

## Data Availability Statement

The raw data supporting the conclusions of this article will be made available by the authors, without undue reservation.

## Ethics Statement

The study and associated protocols were approved by the University of North Texas Health Science Center Institutional Animal Care and Use Committee in Fort Worth, TX prior to initiation of the study.

## Author Contributions

CJ performed the behavior experiments, processed the experimental data, performed the analysis, drafted the manuscript, and designed the figures. CJ, KB, and SS performed preliminary induction experiments. CJ and SS performed gene expression analyses. NS, AG, and KB were involved in planning, supervised the work, aided in interpreting the results, and worked on the manuscript. All authors discussed the results and commented on the manuscript.

## Conflict of Interest

The authors declare that the research was conducted in the absence of any commercial or financial relationships that could be construed as a potential conflict of interest.

## References

[1] Cenker JJ Stultz RD Mcdonald D. Brain microglial cells are highly susceptible to HIV-1 infection and spread. AIDS Res Hum Retrovirus. (2017) 33:1155–65. 10.1089/aid.2017.0004 28486838 PMC5665495

[2] Lee CA Beasley E Sundar K Smelkinson M Vinton C Deleage C. Simian immunodeficiency virus-infected memory CD4^+^ T cells infiltrate to the site of infected macrophages in the neuroparenchyma of a chronic macaque model of neurological complications of AIDS. mBio. (2020) 11:mbio00602. 10.1128/mBio.00602-20 32317323 PMC7175093

[3] Lutgen V Narasipura SD Barbian HJ Richards M Wallace J Razmpour R. HIV infects astrocytes *in vivo* and egresses from the brain to the periphery. PLoS Pathog. (2020) 16:e1008381. 10.1371/journal.ppat.1008381 32525948 PMC7289344

[4] Sanmarti M Ibáñez L Huertas S Badenes D Dalmau D Slevin M HIV-associated neurocognitive disorders. J Mol Psychiatr. (2013) 13:976–86. 10.1186/2049-9256-2-2 PMC4416263 25945248

[5] Eggers C Arendt G Hahn K Husstedt IW Maschke M Neuen-Jacob E. HIV-1-associated neurocognitive disorder: epidemiology, pathogenesis, diagnosis, and treatment. J Neurol. (2017) 264:1715–27. 10.1007/s00415-017-8503-2 28567537 PMC5533849

[6] Sacktor N. Changing clinical phenotypes of HIV-associated neurocognitive disorders. J Neurovirol. (2018) 24:141–5. 10.1007/s13365-017-0556-6 28752495 PMC5787044

[7] Paul R. Neurocognitive phenotyping of HIV in the Era of antiretroviral therapy. Curr HIV AIDS Rep. (2019) 16:230–5. 10.1007/s11904-019-00426-9 31168712 PMC6571160

[8] Gardner J Ghorpade A. Tissue inhibitor of metalloproteinase (TIMP)-1: the TIMPed balance of matrix metalloproteinases in the central nervous system. J Neurosci Res. (2003) 74:801–6. 10.1002/jnr.10835 14648584 PMC3857704

[9] Kaul M Lipton SA. Mechanisms of neuronal injury and death in HIV-1 associated dementia. Curr HIV Res. (2006) 4:307–18. 10.2174/157016206777709384 16842083

[10] Saylor D Dickens AM Sacktor N Haughey N Slusher B Pletnikov M HIV-associated neurocognitive disorder–pathogenesis and prospects for treatment. Nat Rev Neurol. (2016) 12:234–48. 10.1038/nrneurol.2016.27 26965674 PMC4937456

[11] Rumbaugh J Turchan-Cholewo J Galey D St Hillaire C Anderson C Conant K. Interaction of HIV Tat and matrix metalloproteinase in HIV neuropathogenesis: a new host defense mechanism. FASEB J. (2006) 20:1736. 10.1096/fj.05-5619fje 16807369

[12] Louboutin J Reyes B Agrawal L Van Bockstaele E Strayer D. HIV-1 gp120 upregulates matrix metalloproteinases and their inhibitors in a rat model of HIV encephalopathy. Eur J Neurosci. (2011) 34:2015. 10.1111/j.1460-9568.2011.07908.x 22092673

[13] Xu R Feng X Xie X Zhang J Wu D Xu L. HIV-1 Tat protein increases the permeability of brain endothelial cells by both inhibiting occludin expression and cleaving occludin via matrix metalloproteinase-9. Brain Res. (2012) 1436:13. 10.1016/j.brainres.2011.11.052 22197032

[14] Magnoni S Baker A Thomson S Jordan G George SJ Mccoll BW. Neuroprotective effect of adenoviral-mediated gene transfer of TIMP-1 and−2 in ischemic brain injury. Gene Ther. (2007) 14:621–5. 10.1038/sj.gt.3302894 17235293

[15] Tejima E Guo S Murata Y Arai K Lok J Van Leyen K. Neuroprotective effects of overexpressing tissue inhibitor of metalloproteinase TIMP-1. J Neurotrauma. (2009) 26:1935–41. 10.1089/neu.2009.0959 19469687 PMC2822804

[16] Saha P Sarkar S Paidi RK Biswas SC. TIMP-1: a key cytokine released from activated astrocytes protects neurons and ameliorates cognitive behaviours in a rodent model of Alzheimer's disease. Brain Behav Immun. (2020) 87:804–19. 10.1016/j.bbi.2020.03.014 32194232

[17] Ashutosh CC Borgmann K Brew K Ghorpade A. Tissue inhibitor of metalloproteinases-1 protects human neurons from staurosporine and HIV-1-induced apoptosis: mechanisms and relevance to HIV-1-associated dementia. Cell Death Dis. (2012) 3:e332. 10.1038/cddis.2012.54 22739984 PMC3388228

[18] Dzwonek J Rylski M Kaczmarek L. Matrix metalloproteinases and their endogenous inhibitors in neuronal physiology of the adult brain. FEBS Lett. (2004) 567:129–35. 10.1016/j.febslet.2004.03.070 15165905

[19] Nazdik MK Taheri M Sajjadi E Arsang-Jang S Koohpar ZK Inoko H. Increased expression ratio of matrix metalloproteinase-9 (MMP9) and tissue inhibitor of matrix metalloproteinase (TIMP-1) RNA levels in Iranian multiple sclerosis patients. Hum Antibodies. (2016) 24:65–70. 10.3233/HAB-160296 27689613

[20] Boziki M Grigoriadis N. An update on the role of matrix metalloproteinases in the pathogenesis of multiple sclerosis. Med Chem. (2018) 14:155–69. 10.2174/1573406413666170906122803 28875862

[21] Zhang K Mcquibban G Silva C Butler G Johnston J Holden J. HIV-induced metalloproteinase processing of the chemokine stromal cell derived factor-1 causes neurodegeneration. Nat Neurosci. (2003) 6:1064. 10.1038/nn1127 14502291

[22] Eugenin EA Osiecki K Lopez L Goldstein H Calderon TM Berman JW. CCL2/monocyte chemoattractant protein-1 mediates enhanced transmigration of human immunodeficiency virus (HIV)-infected leukocytes across the blood-brain barrier: a potential mechanism of HIV-CNS invasion and NeuroAIDS. J Neurosci. (2006) 26:1098–106. 10.1523/JNEUROSCI.3863-05.2006 16436595 PMC6674577

[23] Xing Y Shepherd N Lan J Li W Rane S Gupta SK. MMPs/TIMPs imbalances in the peripheral blood and cerebrospinal fluid are associated with the pathogenesis of HIV-1-associated neurocognitive disorders. Brain Behav Immun. (2017) 65:161–72. 10.1016/j.bbi.2017.04.024 28487203 PMC5793222

[24] Suryadevara R Holter S Borgmann K Persidsky R Labenz-Zink C Persidsky Y. Regulation of tissue inhibitor of metalloproteinase-1 by astrocytes: Links to HIV-1 dementia. Glia. (2003) 44:47–56. 10.1002/glia.10266 12951656 PMC3820378

[25] Gardner J Borgmann K Deshpande MS Dhar A Wu L Persidsky R. Potential mechanisms for astrocyte-TIMP-1 downregulation in chronic inflammatory diseases. J Neurosci Res. (2006) 83:1281–92. 10.1002/jnr.20823 16555295

[26] Kim BO Liu Y Ruan Y Xu ZC Schantz L He JJ. Neuropathologies in transgenic mice expressing human immunodeficiency virus type 1 Tat protein under the regulation of the astrocyte-specific glial fibrillary acidic protein promoter and doxycycline. Am J Pathol. (2003) 162:1693–707. 10.1016/S0002-9440(10)64304-0 12707054 PMC1851199

[27] Langford D Oh Kim B Zou W Fan Y Rahimain P Liu Y. Doxycycline-inducible and astrocyte-specific HIV-1 Tat transgenic mice (iTat) as an HIV/neuroAIDS model. J Neurovirol. (2018) 24:168–79. 10.1007/s13365-017-0598-9 29143286 PMC6444363

[28] Maggirwar SB Tong N Ramirez S Gelbard HA Dewhurst S. HIV-1 Tat-mediated activation of glycogen synthase kinase-3β contributes to tat-mediated neurotoxicity. J Neurochem. (1999) 73:578–86. 10.1046/j.1471-4159.1999.0730578.x 10428053

[29] Haughey NJ Mattson MP. Calcium dysregulation and neuronal apoptosis by the HIV-1 proteins Tat and gp120. J Acquir Immune Defic Syndr. (2002) 31(Suppl 2):S55–61. 10.1097/00126334-200210012-00005 12394783

[30] Kruman Ii Nath A Mattson MP. HIV-1 protein Tat induces apoptosis of hippocampal neurons by a mechanism involving caspase activation, calcium overload, and oxidative stress. Exp Neurol. (1998) 154:276–88. 10.1006/exnr.1998.6958 9878167

[31] Agrawal L Louboutin J-P Reyes BA Van Bockstaele EJ Strayer DS. HIV-1 Tat neurotoxicity: a model of acute and chronic exposure, and neuroprotection by gene delivery of antioxidant enzymes. Neurobiol Dis. (2012) 45:657–70. 10.1016/j.nbd.2011.10.005 22036626

[32] Theodore S Cass WA Maragos WF. Involvement of cytokines in human immunodeficiency virus-1 protein Tat and methamphetamine interactions in the striatum. Exp Neurol. (2006) 199:490–8. 10.1016/j.expneurol.2006.01.009 16510141

[33] Ju SM Song HY Lee JA Lee SJ Choi SY Park J. Extracellular HIV-1 Tat up-regulates expression of matrix metalloproteinase-9 via a MAPK-NF-kappaB dependent pathway in human astrocytes. Exp Mol Med. (2009) 41:86–93. 10.3858/emm.2009.41.2.011 19287189 PMC2679334

[34] Conant K Garzino-Demo A Nath A Mcarthur J Halliday W Power C. Induction of monocyte chemoattractant protein-1 in HIV-1 Tat-stimulated astrocytes and elevation in AIDS dementia. Proc Natl Acad Sci USA. (1998) 95:3117–21. 10.1073/pnas.95.6.3117 9501225 PMC19704

[35] Fitting S Xu R Bull C Buch SK El-Hage N Nath A. Interactive comorbidity between opioid drug abuse and HIV-1 Tat: chronic exposure augments spine loss and sublethal dendritic pathology in striatal neurons. Am J Pathol. (2010) 177:1397–410. 10.2353/ajpath.2010.090945 20651230 PMC2928972

[36] Carey AN Sypek EI Singh HD Kaufman MJ Mclaughlin JP. Expression of HIV-Tat protein is associated with learning and memory deficits in the mouse. Behav Brain Res. (2012) 229:48–56. 10.1016/j.bbr.2011.12.019 22197678 PMC3580389

[37] Fitting S Scoggins KL Xu R Dever SM Knapp PE Dewey WL. Morphine efficacy is altered in conditional HIV-1 Tat transgenic mice. Eur J Pharmacol. (2012) 689:96–103. 10.1016/j.ejphar.2012.05.029 22659585 PMC3402587

[38] Paris JJ Singh HD Ganno ML Jackson P Mclaughlin JP. Anxiety-like behavior of mice produced by conditional central expression of the HIV-1 regulatory protein, Tat. Psychopharmacology. (2014) 231:2349–60. 10.1007/s00213-013-3385-1 24352568 PMC4020990

[39] Mclaughlin JP Eans S Medina J Hymel K Rock A Mintzopoulos D HIV-1 Tat-protein elevates forebrain glutathione levels and increases morphine drug-seeking and depression-like behaviors in mice. Drug Alcohol Depend. (2017). 171:e139 10.1016/j.drugalcdep.2016.08.386

[40] Kesby JP Chang A Najera JA Marcondes MCG Semenova S. Brain reward function after chronic and binge methamphetamine regimens in mice expressing the HIV-1 TAT protein. Curr HIV Res. (2019) 17:126–33. 10.2174/1570162X17666190703165408 31269883 PMC6995663

[41] Strauss M O'donovan B Ma Y Xiao Z Lin S Bardo MT. [^3^H]Dopamine uptake through the dopamine and norepinephrine transporters is decreased in the prefrontal cortex of transgenic mice expressing HIV-1 transactivator of transcription protein. J Pharmacol Exp Ther. (2020) 374:241–251. 10.1124/jpet.120.266023 32461322 PMC7366287

[42] Nass S Hahn Y Mclane V Varshneya N Damaj M Knapp P. Chronic HIV-1 Tat exposure alters anterior cingulate cortico-basal ganglia-thalamocortical synaptic circuitry, associated behavioral control, and immune regulation in male mice. Brain Behav Immun Health. (2020) 5:77. 10.1016/j.bbih.2020.100077 33083793 PMC7571616

[43] Zhao X Fan Y Vann PH Wong JM Sumien N He JJ. Long-term HIV-1 tat expression in the brain led to neurobehavioral, pathological, and epigenetic changes reminiscent of accelerated aging. Aging Dis. (2020) 11:93–107. 10.14336/AD.2019.0323 32010484 PMC6961778

[44] Shetty RA Forster MJ Sumien N. Coenzyme Q(10) supplementation reverses age-related impairments in spatial learning and lowers protein oxidation. Age. (2013) 35:1821–34. 10.1007/s11357-012-9484-9 23138632 PMC3776107

[45] Chaudhari K Wong JM Vann PH Sumien N Exercise training and antioxidant supplementation independently improve cognitive function in adult male and female GFAP-APOE mice. J Sport Health Sci. (2014) 3:196–205. 10.1016/j.jshs.2014.04.004

[46] Zhou BY Liu Y Kim B Xiao Y He JJ. Astrocyte activation and dysfunction and neuron death by HIV-1 Tat expression in astrocytes. Mol Cell Neurosci. (2004) 27:296–305. 10.1016/j.mcn.2004.07.003 15519244

[47] Bruce-Keller AJ Turchan-Cholewo J Smart EJ Geurin T Chauhan A Reid R. Morphine causes rapid increases in Glia*l* activation and neuronal injury in the striatum of inducible HIV-1 tat transgenic mice. Glia. (2008) 56:1414. 10.1002/glia.20708 18551626 PMC2725184

[48] Hahn YK Podhaizer EM Farris SP Miles MF Hauser KF Knapp PE. Effects of chronic HIV-1 Tat exposure in the CNS: heightened vulnerability of males versus females to changes in cell numbers, synaptic integrity, and behavior. Brain Struc Func. (2015) 220:605. 10.1007/s00429-013-0676-6 24352707 PMC4341022

[49] Fan Y He JJ HIV-1 Tat induces unfolded protein response and endoplasmic reticulum stress in astrocytes and causes neurotoxicity through glial fibrillary acidic protein (GFAP) activation and aggregation. J Biol Chem. (2016) 291:22819–29. 10.1074/jbc.M116.731828 27609520 PMC5077214

[50] Dickens AM Yoo SW Chin AC Xu J Johnson TP Trout AL. Chronic low-level expression of HIV-1 Tat promotes a neurodegenerative phenotype with aging. Sci Rep. (2017) 7:7748. 10.1038/s41598-017-07570-5 28798382 PMC5552766

[51] Fellows RP Byrd DA Morgello S Manhattan HIVBB. Major depressive disorder, cognitive symptoms, and neuropsychological performance among ethnically diverse HIV+ men and women. J Int Neuropsychol Soc. (2013) 19:216–25. 10.1017/S1355617712001245 23290446 PMC3785228

[52] Rivera-Rivera Y Vazquez-Santiago FJ Albino E Sanchez MD Rivera-Amill V. Impact of depression and inflammation on the progression of HIV disease. J Clin Cell Immunol. (2016) 7:423. 10.4172/2155-9899.1000423 27478681 PMC4966661

[53] Paris JJ Fenwick J Mclaughlin JP. Progesterone protects normative anxiety-like responding among ovariectomized female mice that conditionally express the HIV-1 regulatory protein, Tat, in the CNS. Horm Behav. (2014) 65:445–53. 10.1016/j.yhbeh.2014.04.001 24726788 PMC4067900

[54] Crawley JN. Exploratory behavior models of anxiety in mice. Neurosci Biobehav Rev. (1985) 9:37–44. 10.1016/0149-7634(85)90030-2 2858080

[55] Buchanan JB Sparkman NL Chen J Johnson RW. Cognitive and neuroinflammatory consequences of mild repeated stress are exacerbated in aged mice. Psychoneuroendocrinology. (2008) 33:755–65. 10.1016/j.psyneuen.2008.02.013 18407425 PMC2580674

[56] Gehring TV Luksys G Sandi C Vasilaki E. Detailed classification of swimming paths in the morris water maze: multiple strategies within one trial. Sci Rep. (2015) 5:14562. 10.1038/srep14562 26423140 PMC4589698

[57] Cherner M Cysique L Heaton RK Marcotte TD Ellis RJ Masliah E. Neuropathologic confirmation of definitional criteria for human immunodeficiency virus-associated neurocognitive disorders. J Neurovirol. (2007) 13:23–8. 10.1080/13550280601089175 17454445

[58] Fitting S Ignatowska-Jankowska BM Bull C Skoff RP Lichtman AH Wise LE. Synaptic dysfunction in the hippocampus accompanies learning and memory deficits in human immunodeficiency virus type-1 Tat transgenic mice. Biol Psychiatr. (2013) 73:443–53. 10.1016/j.biopsych.2012.09.026 23218253 PMC3570635

[59] Nookala AR Schwartz DC Chaudhari NS Glazyrin A Stephens EB Berman NEJ. Methamphetamine augment HIV-1 Tat mediated memory deficits by altering the expression of synaptic proteins and neurotrophic factors. Brain Behav Immun. (2018) 71:37–51. 10.1016/j.bbi.2018.04.018 29729322 PMC6003882

[60] Hahn YK Paris JJ Lichtman AH Hauser KF Sim-Selley LJ Selley DE. Central HIV-1 Tat exposure elevates anxiety and fear conditioned responses of male mice concurrent with altered mu-opioid receptor-mediated G-protein activation and β-arrestin 2 activity in the forebrain. Neurobiology of disease. (2016) 92:124. 10.1016/j.nbd.2016.01.014 26845176 PMC4907901

[61] Rivera S Garcia-Gonzalez L Khrestchatisky M Baranger K. Metalloproteinases and their tissue inhibitors in Alzheimer's disease and other neurodegenerative disorders. Cell Mol Life Sci. (2019) 76:3167–91. 10.1007/s00018-019-03178-2 31197405 PMC11105182

[62] Jaworski DM. Differential regulation of tissue inhibitor of metalloproteinase mRNA expression in response to intracranial injury. Glia. (2000) 30:199–208. 10.1002/(SICI)1098-1136(200004)30:2<199::AID-GLIA9>3.0.CO;2-# 10719361

[63] Borgmann K Ghorpade A. HIV-1, methamphetamine and astrocytes at neuroinflammatory Crossroads. Front Microbiol. (2015) 6:1143. 10.3389/fmicb.2015.01143 26579077 PMC4621459

[64] Atwood WJ Tornatore CS Meyers K Major EO. HIV-1 mRNA transcripts from persistently infected human fetal astrocytes. Ann N Y Acad Sci. (1993) 693:324–5. 10.1111/j.1749-6632.1993.tb26298.x 8267293

[65] Thompson KA Mcarthur JC Wesselingh SL. Correlation between neurological progression and astrocyte apoptosis in HIV-associated dementia. Ann Neurol. (2001) 49:745–52. 10.1002/ana.1011 11409426

[66] Churchill MJ Wesselingh SL Cowley D Pardo CA Mcarthur JC Brew BJ. Extensive astrocyte infection is prominent in human immunodeficiency virus-associated dementia. Ann Neurol. (2009) 66:253–8. 10.1002/ana.21697 19743454

[67] Saito Y Sharer LR Epstein LG Michaels J Mintz M Louder M. Overexpression of nef as a marker for restricted HIV-1 infection of astrocytes in postmortem pediatric central nervous tissues. Neurology. (1994) 44:474–81. 10.1212/WNL.44.3_Part_1.474 8145918

[68] Chauhan A Turchan J Pocernich C Bruce-Keller A Roth S Butterfield DA. Intracellular human immunodeficiency virus Tat expression in astrocytes promotes astrocyte survival but induces potent neurotoxicity at distant sites via axonal transport. J Biol Chem. (2003) 278:13512–9. 10.1074/jbc.M209381200 12551932

[69] Edara VV Ghorpade A Borgmann K Insights into the gene expression profiles of active and restricted Red/Green-HIV^+^ human astrocytes: implications for shock or lock therapies in the brain. J Virol. (2020) e01563–19. 10.1128/JVI.01563-19 31896591 PMC7158706

[70] Nath A Conant K Chen P Scott C Major EO. Transient exposure to HIV-1 Tat protein results in cytokine production in macrophages and astrocytes. A hit and run phenomenon. J Biol Chem. (1999) 274:17098–102. 10.1074/jbc.274.24.17098 10358063

[71] Leveque T Le Pavec G Boutet A Tardieu M Dormont D Gras G. Differential regulation of gelatinase A and B and TIMP-1 and−2 by TNFalpha and HIV virions in astrocytes. Microbes Infect. (2004) 6:157–63. 10.1016/j.micinf.2003.11.006 14998513

[72] Dhar A Gardner J Borgmann K Wu L Ghorpade A. Novel role of TGF-beta in differential astrocyte-TIMP-1 regulation: implications for HIV-1-dementia and neuroinflammation. J Neurosci Res. (2006) 83:1271–80. 10.1002/jnr.20787 16496359 PMC3820372

[73] Buscemi L Ramonet D Geiger JD. Human immunodeficiency virus type-1 protein tat induces tumor necrosis factor-α-mediated neurotoxicity. Neurobiol Dis. (2007) 26:661. 10.1016/j.nbd.2007.03.004 17451964 PMC2080622

[74] Brew K Dinakarpandian D Nagase H. Tissue inhibitors of metalloproteinases: evolution, structure and function. Biochim Biophys Acta. (2000) 1477:267–83. 10.1016/S0167-4838(99)00279-4 10708863

[75] Visse R Nagase H. Matrix metalloproteinases and tissue inhibitors of metalloproteinases: structure, function, and biochemistry. Circ Res. (2003) 92:827. 10.1161/01.RES.0000070112.80711.3D 12730128

[76] Waubant E Goodkin DE Gee L Bacchetti P Sloan R Stewart T. Serum MMP-9 and TIMP-1 levels are related to MRI activity in relapsing multiple sclerosis. Neurology. (1999) 53:1397–401. 10.1212/WNL.53.7.1397 10534241

[77] Waubant E Goodkin D Bostrom A Bacchetti P Hietpas J Lindberg R. IFNbeta lowers MMP-9/TIMP-1 ratio, which predicts new enhancing lesions in patients with SPMS. Neurology. (2003) 60:52–7. 10.1212/WNL.60.1.52 12525717

[78] Kurzepa J Szczepanska-Szerej A Stryjecka-Zimmer M Malecka-Massalska T Stelmasiak Z. Simvastatin could prevent increase of the serum MMP-9/TIMP-1 ratio in acute ischaemic stroke. Folia Biol. (2006) 52:181−3. Available online at: https://fb.cuni.cz/Data/files/folia_biologica/volume_52_2006_6/FB2006A0023.pdf 17184595 10.14712/fb2006052060181

[79] Li DD Song JN Huang H Guo XY An JY Zhang M. The roles of MMP-9/TIMP-1 in cerebral edema following experimental acute cerebral infarction in rats. Neurosci Lett. (2013) 550:168–72. 10.1016/j.neulet.2013.06.034 23819982

[80] Ridnour LA Dhanapal S Hoos M Wilson J Lee J Cheng RY. Nitric oxide-mediated regulation of beta-amyloid clearance via alterations of MMP-9/TIMP-1. J Neurochem. (2012) 123:736–49. 10.1111/jnc.12028 23016931 PMC3614913

[81] Burggraf D Trinkl A Dichgans M Hamann G. Doxycycline inhibits MMPs via modulation of plasminogen activators in focal cerebral ischemia. Neurobiol Dis. (2007) 25:506. 10.1016/j.nbd.2006.10.013 17166729

[82] Li S Wu Y Keating SM Du H Sammet CL Zadikoff C. Matrix metalloproteinase levels in early HIV infection and relation to *in vivo* brain status. J Neurovirol. (2013) 452–60. 10.1007/s13365-013-0197-3 23979706 PMC3819028

[83] Jourquin J Tremblay E Bernard A Charton G Chaillan FA Marchetti E. Tissue inhibitor of metalloproteinases-1 (TIMP-1) modulates neuronal death, axonal plasticity, and learning and memory. Eur J Neurosci. (2005) 22:2569–78. 10.1111/j.1460-9568.2005.04426.x 16307599

[84] Chaillan FA Rivera S Marchetti E Jourquin J Werb Z Soloway PD. Involvement of tissue inhibition of metalloproteinases-1 in learning and memory in mice. Behav Brain Res. (2006) 173:191–8. 10.1016/j.bbr.2006.06.020 16860884 PMC2659720

[85] Mizoguchi H Ibi D Takuma K Toth E Sato J Itohara S Alterations of emotional and cognitive behaviors in matrix metallo-proteinase-2 and-9-deficient mice. Open Behav Sci J. (2010) 4:19–25. 10.2174/1874230001004010019

[86] Nagy V Bozdagi O Huntley GW. The extracellular protease matrix metalloproteinase-9 is activated by inhibitory avoidance learning and required for long-term memory. Learn Memory. (2007) 14:655. 10.1101/lm.678307 17909100 PMC2044557

[87] Okulski P Jay T Jaworski J Duniec K Dzwonek J Konopacki F. TIMP-1 abolishes MMP-9-dependent long-lasting long-term potentiation in the prefrontal cortex. Biol Psychiatr. (2007) 62:359. 10.1016/j.biopsych.2006.09.012 17210139

[88] Sacktor N Skolasky RL Seaberg E Munro C Becker JT Martin E. Prevalence of HIV-associated neurocognitive disorders in the multicenter AIDS cohort study. Neurology. (2016) 86:334–40. 10.1212/WNL.0000000000002277 26718568 PMC4776086

[89] Atluri VSR Jayant RD Pilakka-Kanthikeel S Garcia G Samikkannu T Yndart A. Development of TIMP1 magnetic nanoformulation for regulation of synaptic plasticity in HIV-1 infection. Int J Nanomed. (2016) 11:4287. 10.2147/IJN.S108329 27621622 PMC5012635

[90] Joshi CR Raghavan V Vijayaraghavalu S Gao Y Saraswathy M Labhasetwar V. Reaching for the stars in the brain: polymer-mediated gene delivery to human astrocytes. Mol Ther Nucleic Acids. (2018) 12:645–57. 10.1016/j.omtn.2018.06.009 30081235 PMC6082920

[91] Proulx J Joshi C Vijayaraghavalu S Saraswathy M Labhasetwar V Ghorpade A. Arginine-modified polymers facilitate poly (lactide-co-glycolide)-based nanoparticle gene delivery to primary human astrocytes. Int J Nanomed. (2020) 15:3639–47. 10.2147/IJN.S250865 32547019 PMC7250304

