# Supplementary material for: Astrocyte HIV-1 Tat Differentially Modulates Behavior and Brain MMP/TIMP Balance During Short and Prolonged Induction in Transgenic Mice
Source: Front Neurol. 2020 Dec 15;11:593188. doi: 10.3389/fneur.2020.593188 (PMC7769877; doi:10.3389/fneur.2020.593188)
Supplement: Supplementary file 1 [file Presentation_1.pdf]

Supplementary Material

Supplementary Figure 1.

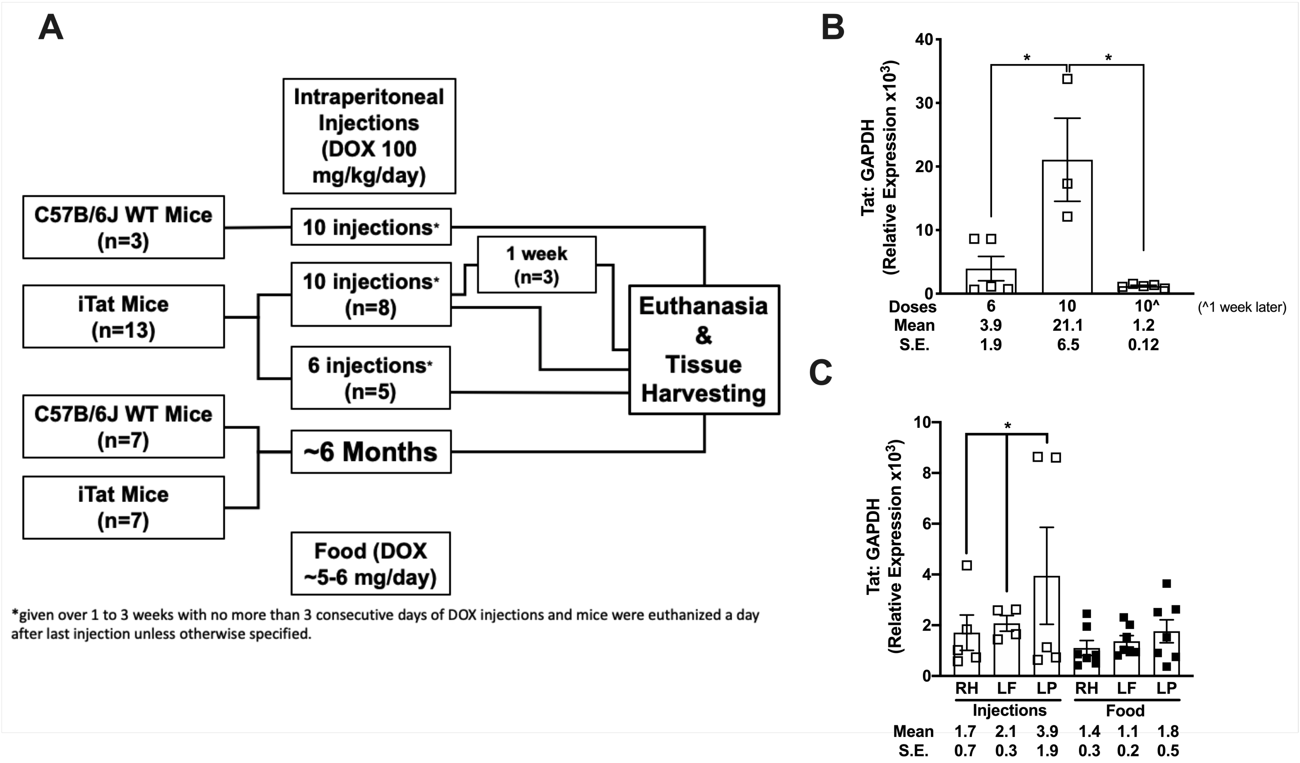

Spatio-temporal changes in HIV-1 Tat expression depict distinct patterns. (A) C57BL/6J wild-type (WT) and iTat mice of both sexes were administered doxycycline (DOX) *via* intraperitoneal (*i.p.*) injections (100 mg/kg/day) or DOX-containing food *ad libitum* (about 5 – 6 mg DOX/day). Mice were euthanized, and brain tissues were harvested for RNA isolation followed by gene expression analyses. Tat mRNA expression was measured in iTat mice using one-step real-time PCR. GAPDH was used as an internal housekeeping control. (**B**) The iTat mice received acute Tat induction by different frequencies of DOX *i.p.* injections at a single 100 mg/kg/day dose (open squares), and brains were harvested at different times after injections including a day after six injections (n=5), a day after ten injections (n=3), and a week after ten injections (n=5). Each bar represents the mean ± SEM. *p<0.05 by one-way ANOVA. (**C**) The iTat mice received acute Tat induction by DOX *i.p.* injections (6 injections, n=5, open squares) or a prolonged Tat induction *via* DOX food (n=7, solid squares). Brains were harvested from mice, and RNA was isolated as three separate fractions including right hemisphere (RH), left forebrain (LF) and left posterior (LP). Relative gene expression of Tat was measured. Each bar represents the mean ± SEM. *p<0.050 by one-way ANOVA.

Supplementary Figure 2.

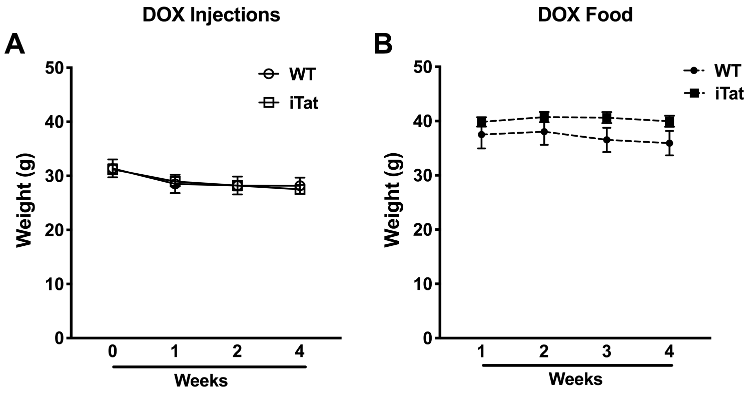

Animal weights for iTat mice were comparable to Treatment Time WT Controls. Weights were measured weekly in both WT and iTat mice that were (**A**) injected or (**B**) fed DOX. **(A)** The WT mice (n = 7, solid line with open circles) and the iTat mice (n = 11, solid line with open squares) received acute Tat induction by DOX *i.p.* injections, (**B**) while the WT mice (n = 13, dashed line with solid circles) and the iTat mice (n = 13, dashed line with solid squares) received prolonged Tat induction *via* DOX food. Each data point represents the mean ± SEM. Data not significant.

Supplementary Figure 3.

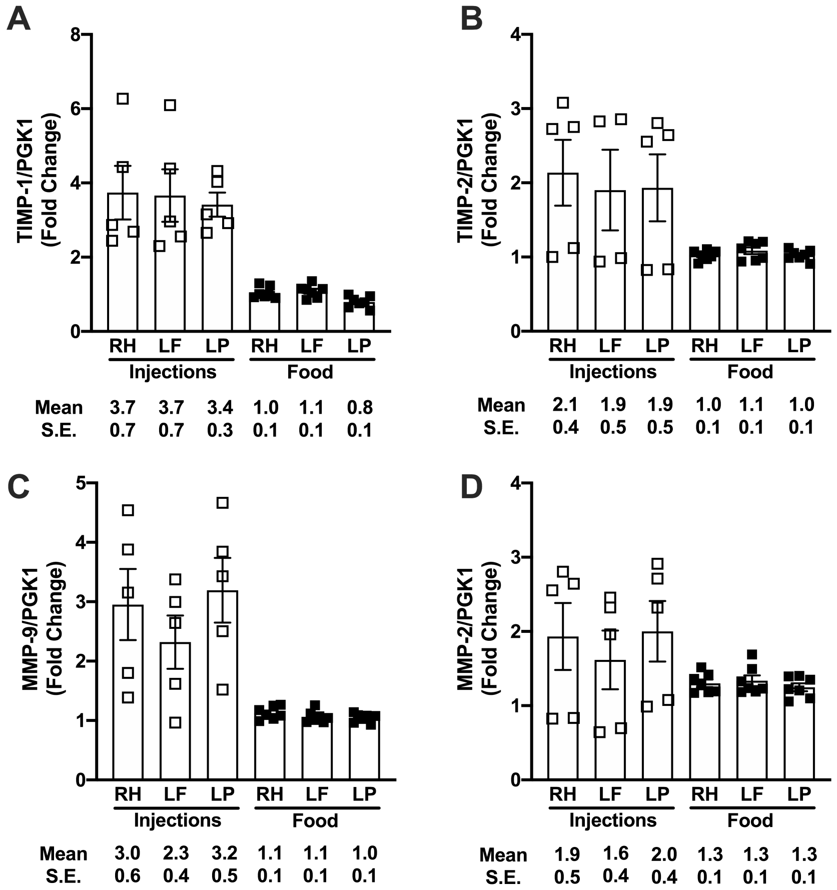

Relative gene expression of the MMP/TIMP axis components remain comparable in different gross brain regions. (**A**) TIMP-1**,** (**B**) TIMP-2**, (C)** MMP-9**, (D)** and MMP-2 gene expression was measured in iTat mice. The iTat mice received acute Tat induction by DOX *i.p.* injections (6 injections, n=5, open squares) or a prolonged Tat induction *via* DOX food (n=7, solid squares). In parallel, WT mice were used for both Treatment Time paradigms (n=3 for injections and n=7 for food). Brains were harvested from mice, and RNA was isolated as three separate fractions including right hemisphere (RH), left forebrain (LF) and left posterior (LP). The relative changes in Gene expression was obtained by normalized against gene expression in WT control mice (not shown). Each data point represents the mean ± SEM. There were no significant differences between brain regions for the same Treatment paradigm.

Supplementary Table 1. The C_t values for commonly used housekeeping genes GAPDH, β-actin, PGK-1.

	GAPDH C_t Value		β-Actin C_t Value		PGK-1 C_t Value	
**Strain/Gene**	**Mean**	**SD**	**Mean**	**SD**	**Mean**	**SD**
**WT**	16.74	0.11	18.28	0.34	20.94	0.12
**iTat**	17.37	0.11	18.14	0.39	20.93	0.29

Mean and standard deviations of GAPDH, β-actin, and pGK-1 C_t values are presented from DOX-Fed WT (n=13) and iTat (n=13) mice. Each value used to calculate the mean was an average of triplicates measurements per sample.
